# Food insecurity in the Eastern Indo-Gangetic plain: Taking a closer look

**DOI:** 10.1371/journal.pone.0279414

**Published:** 2023-01-05

**Authors:** Saumyadipta Pyne, Saurav Guha, Sumonkanti Das, Meghana Ray, Hukum Chandra

**Affiliations:** 1 Department of Statistics and Applied Probability, University of California Santa Barbara, Santa Barbara, California, United States of America; 2 Health Analytics Network, Pittsburgh, Pennsylvania, United States of America; 3 ICAR-Indian Agricultural Statistics Research Institute, New Delhi, India; 4 School of Demography, Australian National University, Canberra, Australia; University of Pisa, ITALY

## Abstract

**Objective:**

Food security is an important policy issue in India. As India recently ranked 107^th^ out of 121 countries in the 2022 Global Hunger Index, there is an urgent need to dissect, and gain insights into, such a major decline at the national level. However, the existing surveys, due to small sample sizes, cannot be used directly to produce reliable estimates at local administrative levels such as districts.

**Design:**

The latest round of available data from the Household Consumer Expenditure Survey (HCES 2011–12) done by the National Sample Survey Office of India used stratified multi-stage random sampling with districts as strata, villages as first stage and households as second stage units.

**Setting:**

Our Small Area Estimation approach estimated food insecurity prevalence, gap, and severity of each rural district of the Eastern Indo-Gangetic Plain (EIGP) region by modeling the HCES data, guided by local covariates from the 2011 Indian Population Census.

**Participants:**

In HCES, 5915 (34429), 3310 (17534) and 3566 (15223) households (persons) were surveyed from the 71, 38 and 18 districts of the EIGP states of Uttar Pradesh, Bihar and West Bengal respectively.

**Results:**

We estimated the district-specific food insecurity indicators, and mapped their local disparities over the EIGP region. By comparing food insecurity with indicators of climate vulnerability, poverty and crop diversity, we shortlisted the vulnerable districts in EIGP.

**Conclusions:**

Our district-level estimates and maps can be effective for informed policy-making to build local resiliency and address systemic vulnerabilities where they matter most in the post-pandemic era.

**Advances:**

Our study computed, for the Indian states in the EIGP region, the first area-level small area estimates of food insecurity as well as poverty over the past decade, and generated a ranked list of districts upon combining these data with measures of crop diversity and climatic vulnerability.

## 1. Introduction

Food security exists when all people, at all times, have physical and economic access to sufficient, safe and nutritious food that meets their dietary needs and food preferences for an active and healthy life, according to Food and Agriculture Organization (FAO) [1]. Conversely, food insecurity (FI) refers to a situation wherein members of society have “limited access to safe and healthy food” [2]. In the absence of food security, nutritional deficits may lead to stunting, wasting, and undernourishment that bring forth a sequalae of developmental, socioeconomic, and health consequences that impact societies in both short and long terms. In fact, it is believed that there can be no environmental security and sustainability without food security [3].

Over the past 50 years, gains in agricultural productivity from the Green Revolution have translated to increased food availability [4]. With about six-fold increase in food grain production from 50 million tonnes in 1950–1951 to about 291.95 million tonnes in 2019–2020, India has moved away from dependence on food aid to become a net food exporter country. Yet, it faces a complex challenge from multiple burdens of undernutrition, micronutrient deficiency, as well as obesity [5]. Recently, India ranked 107th among 121 countries in the 2022 Global Hunger Index (GHI) report (although this was disputed by its Government) [6]. Among Indian children of age 5 years and younger, 34.7% were stunted, 17.3% wasted, and 33.4% underweight; and among children of age 5–9 years, 21.9% were stunted, and 23% moderately-to-severely thin for their age [7]. More than half the women of reproductive age (15–49 years) in India are anemic while studies on long-term effects of early-life and pre-natal hunger among Indian populations are underway [8].

Research on climate change over the past decade has revealed the importance of climate pollutants (both short- and long-term) on monsoon patterns, precipitation changes, and increases in temperature [9]. A 15–30% decline in production of most cereals has been projected for Africa and South Asia [10]. In India, rice and wheat contribute to three-quarters of the country’s cereal production and their yields are susceptible to climate changes. Rainfed agriculture supports about 40% of India’s population and estimates of the past data show that changes in monsoon characteristics led to reduced rice yields in India by 1.7% during 1960–2002 [9]. For every 1-degree Celsius increase in temperature, loss of 3.7%-14.5% in India’s wheat yields was estimated. For rice, such estimates from multiple methods predict even larger temperature impact with an average reduction of 6.6 ± 3.8% per degree Celsius [11].

To study the complex interplay among socioeconomic conditions, agroecology, and climate change, and their combined effects on food security, few regions are as crucially important as India’s “breadbasket”, the Indo-Gangetic plain. The region comprises of a 2.5 million km^2^ fertile plain encompassing the northern regions of the Indian subcontinent. In particular, the Eastern Indo-Gangetic Plains (EIGP) region includes the states of Uttar Pradesh (UP), Bihar, and West Bengal (WB) in India, as well as parts of Nepal and Bangladesh. In India, EIGP consists of 39.27 million hectares and is home to 395.19 million people, i.e., 32.64% of India’s total population (2011 census). It is among the most densely populated (700–1200 persons/km^2^) regions in the world, and has high socioeconomic vulnerability.

EIGP is characterized by fertile soils with ample monsoon rainfall, continuous supply of surface and groundwater and a largely favorable climate that supports a predominantly rice-wheat cropping pattern. While UP and Bihar account for 32% and 5.76% of the country’s total wheat production respectively, WB, Bihar, and UP contribute respectively 13.26%, 7%, and 11.75% of its total rice production [12]. However, EIGP’s food security is potentially vulnerable to adverse effects of both anthropogenic and environmental factors. In 2012, the percentage of populations living below the national poverty line for UP, Bihar, and WB was 29.43, 33.74, and 19.98 respectively [13]. The rates of unemployment and seasonal out-migration are relatively high among its rural populations while land holdings are generally small.

EIGP’s environmental concerns include rising temperatures, high inter-annual variability of precipitation and frequent occurrence of adverse climatic events such as droughts, floods, and increasing cyclonic activity [14, 15]. Annual buildups of atmospheric pollutants in intensively farmed areas may have resulted in relative yield changes of -15% or greater in this region between 2006 and 2010 [16]. The Ganga basin also has severe groundwater contamination of Arsenic, which enters the food chain, especially through cultivation of rice [17]. In fact, disproportionately large contribution of rice production to resource use, greenhouse gases, and climate sensitivity (relative to its share of monsoon cereal calorie production) was observed in India [18]. Studies have noted the importance of addressing the impacts of climate change through solutions such as diet and crop diversification, improved farming technology, issues of governance, and other adaptations [19, 20].

Given the region’s diverse agroecological and biophysical conditions, availability of precise and timely disaggregate level statistics is essential for designing targeted policies to ensure food security in EIGP. In developing countries, however, the scarcity of reliable quantitative data can present a major challenge to policy-makers and researchers [21]. Such data, even when they exist, are often reported only at regional or state levels, and may be poorly correlated with local surveys [22]. In particular, FI presents a complex systemic challenge to policy planners, researchers, and government agencies at different administrative levels. To understand it closely, it is essential to disaggregate nationally-representative FI data for small geographic areas (e.g., districts) or demographic groups (e.g., gender-wise, social groups). However, in large-scale survey data, the sample sizes of such small areas may be very small or even zero. Small area estimation (SAE) provides us with an efficient approach to address this challenge with precision and rigor [23].

While there is no single universally accepted measure of food insecurity (FI), some classes of FI indicators are common in policy literature [24, 25]. A comparative performance analysis of the FAO estimates with anthropometric measures for nutritional assessment and estimates from household consumption expenditure surveys (HCESs) showed that the latter apparently has the most benefits among FI indicators [24]. HCESs can offer a less expensive yet more regularly-conducted means of detailed dietary intake assessment, especially for some developing countries. HCESs usually capture data on a detailed breakdown of household food expenditures, including food quantities and associated monetary transfers. This allows disaggregation of FI by geographic areas or socioeconomic groups, thus revealing any inequalities of food consumption within a country. A nationally representative HCES can reveal the share of households in the sample falling under a fixed nutritional threshold, and thus, provide an estimate of the prevalence of undernourishment in the country [24]. We captured this with the indicator FI Prevalence (FIP). Further, some indicators are more sensitive at certain severity levels [25] of FI, which are captured by the indicators FI Gap and Severity (FIG and FIS) and used in the present as well as past studies [26].

In rural India, FI is based on the average calorie intake of less than 2400 kcal per capita per day, a threshold based on the direct calorie intake method [27]. While small area estimates of FIP, FIG and FIS were recently computed for Bangladesh [26], no such FI data exist for the much larger, more populous and diverse EIGP region that lies within India. The HCES data are collected by the National Sample Survey Office (NSSO), Ministry of Statistics and Program Implementation, Government of India. However, researchers have noted the lack of publication of such data by the government after the 68^th^ round of HCES in 2011–2012 [28]. However, while such nationwide surveys provide reliable state and national level estimates, they cannot directly produce reliable estimates at a lower administrative level, such as a district, due to small sample sizes. Therefore, they cannot reveal the existing local disparities in food security among the different populations living in hundreds of districts, especially in rural India.

The present study has several distinctive and innovative features. In this study, we used SAE to compute model-based district level estimates of FI prevalence (FIP), gap (FIG) and severity (FIS) in EIGP region covering the rural parts of the Indian states of UP, Bihar, and WB based on HCES 2011–2012 data and informed by local covariates. Previous small area studies [29, 30] have either estimated different outcome variables such as *nutritional intake* (protein, fat, etc.) or used *area level* models. In contrast, the present study is based on a more detailed small area model that allows us to estimate the measures of FI using *individual level* survey data by incorporating household level survey weight. Moreover, while the past studies [29, 30] have taken an isolated view of only one state, we have considered EIGP as a connected *region* known for its unique demographics, agroecology, and hydroclimatic risks. Thus, we included key regional parameters such as climatic vulnerability, crop diversity, poverty, and the disparities of FI across the Gangetic and non-Gangetic districts. This makes the present study distinctive in terms of its broader geographic scope, different model assumptions and outcome variables, and estimation of multiple additional district-specific indices.

The data used for SAE, and the SAE methodology of this study, are described in the next section. In Section 3, SAE results are discussed in detail while different maps shed light on the district-level disparities of food security in the EIGP region. Further, by comparison of FI with other indicators such as climate vulnerability, poverty, and crop diversity, we provide a shortlist of districts that could be useful for future studies. We end with concluding remarks in Section 4.

## 2. Materials and methods

### 2.1 SAE data

We used the latest round of available HCES in India, i.e., HCES 2011–2012, for rural areas of EIGP region, including UP, Bihar and WB. Usually, over the duration of a year, these surveys are conducted through interviews of a representative sample of households. The sampling design used is stratified multi-stage random sampling with districts as strata, villages as first stage units and households as second stage units. In this survey, a total of 12,791 households and 67,186 persons were surveyed from 127 districts of the EIGP region. In particular, 5915 (34429), 3310 (17534) and 3566 (15223) households (persons) were surveyed from 71, 38 and 18 districts of UP, Bihar and WB respectively. This study analyzed publicly available, deidentified and anonymized data. Data for analyses are within the manuscript and its Supporting Information file. The de-identified survey data are available from the National Sample Survey Office, Government of India (Website: http://mospi.nic.in/).

The 2011–2012 HCES provides information on the quantity and value of more than 142 food items with a reference period of last 30 days for a few food items and last 7 days for the remaining food items for each state separately for rural and urban areas. The state-level estimates of average calorie intake for UP, Bihar, WB and EIGP are 2200, 2242, 2199 and 2212 kcal per capita per day respectively. The quantities of food recorded as consumed by the household were converted into equivalent amounts of energy based on a nutrition chart, that provides the energy per unit of different foods in the Indian diet [31]. Note, our analyses and outputs cover all the rural districts surveyed in this HCES, some of which have since been split to form newer districts. Kolkata, an urban district and the capital city of WB, was excluded from the rural districts of EIGP.

The auxiliary variables taken from the 2011 Population Census of India [32] are available as counts at district level. We fit models with the direct survey estimates of FI indicators as the response variables and a selected set of 5 auxiliary variables as potential covariates: (1) Number of households (HH), (2) Proportion of scheduled caste population (SC), (3) Proportion of scheduled tribe population (ST), (4) Literacy rate (LR), and (5) Proportion of working population (WP). For our analysis, we used the average dietary intake in rural India to be 2400 kcal per capita per day, as recommended by the Ministry of Health and Family Welfare, Government of India [33].

### 2.2 District level poverty, crop diversity, and climate vulnerability

We used the same HCES data for estimating district-level measure of poverty given by a poverty index (PI) for UP, Bihar and WB as described in S1 File. We computed a district-specific entropy-based crop diversity index (CDI) based on land use statistics from the Ministry of Agriculture and Farmers’ Welfare, Government of India [34]. The CDI calculation is described in the S1 File. Recently, a report on the detailed national-scale assessment of climate vulnerability was released by the Department of Science & Technology (DST), Government of India; and the Swiss Agency for Development & Cooperation (SDC). The DST-SDC report [14] lists all districts in India ranked by their Climate Vulnerability Index (CVI), which we used for our analysis.

### 2.3 Measures of food insecurity

The target variable *Y* at the unit (household) level in the 2011–12 HCES survey data file is binary, corresponding to whether a household is “food insecure” (i.e., consumes less than 2400 Kcal per capita per day in rural India) or not. Let *U* denote the finite population of interest of size *N* partitioned into *D* disjoint small areas (here, districts), a sample *s* of size *n* is drawn from this population with a given survey design. Let the set of population units i.e., households in area *i*(*i* = 1, …, *D*) be denoted by *U*_*i*_ with known size *N*_*i*_ (i.e., the number of households in district *i*) such that $U=\cup_{i=1}^{D}U_{i}$ and $N=\sum_{i=1}^{D}N_{i}$. Let *s*_*i*_ denote the collection of units making up the sample in area *i* such that $s=\cup_{i=1}^{D}s_{i}$ and $n=\sum_{i=1}^{D}n_{i}$. Let *y*_*ij*_ denote the value of the variable of interest for unit *j*(*j* = 1, …, *N*_*i*_) in area *i*. In this study, *y*_*ij*_ is the daily per capita calorie intake (averaged at the household level) for household *j* in district *i*.

### 2.4 FIP, FIG and FIS

The quantity that we are interested to estimate for area (i.e., district) *i* is the food insecurity indicator *F*_*αi*_ [35] defined as

$$F_{\alpha i}=N_{i}^{-1}\sum_{j\in U_{i}}F_{\alpha ij},$$ (1)

where *F*_*αij*_ = (1 − *y*_*ij*_/*z*)^*α*^ *I*(*y*_*ij*_ ≤ *z*) and *z* is a preset threshold value of food insecurity (*z* = 2400 kcal in this study), *I*(*y*_*ij*_ ≤ *z*) indicator variable for a food insecure household *j* in district *i* and *α* is “sensitivity” parameter. The FI indicators FIP, FIG and FIS are calculated by setting *α* = 0, 1 and 2 respectively in [1].

### 2.5 SAE methodology

When unit level covariates are available, unit level SAE methodology dedicated to poverty estimation [36–38] can be utilized to estimate food insecurity indicators. Since district-specific covariates extracted from full census data are available, area level SAE methods were implemented in this study. Fay–Herriot model [39] is a widely used area level method in SAE that assumes the availability of area-specific survey estimates, and that these follow a linear mixed model with area random effects. We used the Fay–Herriot model [39] to develop district level estimates of FIG and FIS. To compute model-based district level estimates of FIP, we modeled survey weighted area-specific proportions under a logistic linear mixed model (LLMM) [40]. In this study, a district (of EIGP) is considered as the small area of interest.

### 2.6 The direct estimator

Without using any auxiliary data, this estimator, denoted by Direct, of *F*_*αi*_ is ${\hat{F}}_{\alpha i}^{Direct\;}={\left(\sum_{j\in s_{i}}w_{ij}\right)}^{-1}\sum_{j\in s_{i}}w_{ij}F_{\alpha ij}$, where *w*_*ij*_ denotes the survey weights associated with unit *j* in district *i* [21]. For *α* = 0, $p_{iw}={\hat{F}}_{0i}={\left(\sum_{j\in s_{i}}w_{ij}\right)}^{-1}\sum_{j\in s_{i}}w_{ij}I\left(y_{ij}\leq z\right)$ defines the survey weighted direct estimator of the proportion of food insecure households in district *i*.

The variance of the direct estimator ${\hat{F}}_{\alpha i}^{Direct\;}$ is approximated by $\psi_{\alpha i}=var\left({\hat{F}}_{\alpha i}^{Direct\;}\right)\approx {\left(\sum_{j\in s_{i}}w_{ij}\right)}^{-2}\sum_{j\in s_{i}}w_{ij}\left(w_{ij}-1\right){\left(F_{\alpha ij}-{\hat{F}}_{\alpha i}^{Direct\;}\right)}^{2}$. Note, when a district *i*’s sample size is small, the sampling variance *ψ*_*αi*_ is large and hence the direct estimator could be unstable due to large standard error.

### 2.7 SAE of FIG and FIS

Let ${\hat{F}}_{\alpha i}^{Direct\;}$ be the observed direct estimate of unobservable population parameter *F*_*αi*_ of variable of interest *y* for district *i*. Let **x**_*i*_ be the *k*-vector of district level auxiliary variables, often obtained from various administrative and census records, related to the population parameter *F*_*αi*_. The simple district-specific 2-stage Fay and Herriot model [39] is described as ${\hat{F}}_{\alpha i}^{Direct\;}=F_{\alpha i}+e_{\alpha i}$ and $F_{\alpha i}={\text{x}}_{i}^{T}\beta +u_{i}$. Alternatively, we can express it as

$${\hat{F}}_{\alpha i}^{Direct\;}={\text{x}}_{i}^{T}\beta +u_{i}+e_{\alpha i};\;i=1,\ldots ,D.$$ (2)

Here **β** is a *k*-vector of unknown fixed effects parameters, *u*_*i*_’*s* are independently and identically distributed normal random errors with *E*(*u*_*i*_) = 0 and $Var\left(u_{i}\right)=\sigma_{u}^{2}$, and *e*_*αi*_’s are independent sampling errors normally distributed with *E*(*e*_*αi*_|*F*_*αi*_) = 0, *Var*(*e*_*αi*_|*F*_*αi*_) = *ψ*_*αi*_. The two errors are independent of each other within and across districts. Usually, the sampling variance *ψ*_*αi*_ are assumed to closely approximate the unknown sampling variances, and in some cases, they are smoothed when they are too noisy due to very small sample size. The $\sigma_{u}^{2}$ is unknown and needs to be estimated. Methods of estimating $\sigma_{u}^{2}$ include maximum likelihood (ML) and restricted maximum likelihood (REML) under normality assumptions. Let ${\hat{\sigma }}_{u}^{2}$ denote the resulting estimator of $\sigma_{u}^{2}$ and $\hat{\beta }$ the empirical best linear unbiased estimator of **β**. Then under (2), the empirical best linear unbiased predictor (EBLUP) estimate of *F*_*αi*_ is

$${\hat{F}}_{\alpha i}^{EBLUP}={\text{x}}_{i}^{T}\hat{\beta }+{\hat{u}}_{i};\;i=1,\ldots ,D.$$ (3)

Here, ${\hat{u}}_{i}={\hat{\gamma }}_{i}\left({\hat{F}}_{\alpha i}^{Direct\;}-{\text{x}}_{i}^{T}\hat{\beta }\right)$, where ${\hat{\gamma }}_{i}={\hat{\sigma }}_{u}^{2}{\left({\hat{\sigma }}_{u}^{2}+\psi_{\alpha i}\right)}^{-1}$ defines the shrinkage effect for district *i*. The mean squared error (MSE) estimation of EBLUP follows from Rao and Molina (2015) [23].

### 2.8 SAE of FIP

Estimation of small area proportions using such linear mixed models for continuous data, and derived with EBLUP, might be inconsistent in the sense that the prediction might not be within the [0,1] interval and a suitable transformation (for example, logarithm, square-root, arcsin) is needed for making the prediction within the [0–1] interval with the cost of bias correction for back transformation by utilizing the sampling error variances (noisy variances may lead to worse bias correction) [41, 42]. Thus, the small area proportions are assumed to follow a LLMM approach [23, 40] to produce model-based district level estimates of FIP. Let $I_{si}=\sum_{j\in s_{i}}I\left(y_{ij}\leq z\right)$ denote the sample count (e.g., number of food insecure households in a sample) in district *i*. If the sampling design is ignored and the number of food insecure households are independently and identically distributed within a district, then the sample count in district *i* can be assumed to follow a Binomial distribution with parameters *n*_*i*_ and *π*_*i*_, i.e., *I*_*si*_|*v*_*i*_ ~ *Bin*(*n*_*i*_, *π*_*i*_), where *π*_*i*_ is success probability [40]. Following Johnson et al. (2010) [43] and Chandra et al. (2011) [44], the model linking the probability *π*_*i*_ with the covariates **x**_*i*_ is the LLMM of form

$$logit\left(\pi_{i}\right)=\text{ln}\left\{\pi_{i}{\left(1-\pi_{i}\right)}^{-1}\right\}=\eta_{i}={\text{x}}_{i}^{T}\beta +v_{i},$$ (4)

i.e., $\pi_{i}=\text{exp}\left({\text{x}}_{i}^{T}\beta +v_{i}\right){\left\{1+\text{exp}\left({\text{x}}_{i}^{T}\beta +v_{i}\right)\right\}}^{-1}$. Here *v*_*i*_ is the area-specific random effect that capture the area dissimilarities with $v_{i}\sim N\left(0,\sigma_{v}^{2}\right)$ and **β** is the *k*-vector of regression coefficients, often known as fixed effects parameters. Under (3), a plug-in empirical predictor of proportion (EPP) (e.g., FIP) *F*_0*i*_ in district *i* is

$${\hat{F}}_{0i}^{EPP}=\text{exp}\left({\text{x}}_{i}^{T}\hat{\beta }+{\hat{v}}_{i}\right){\left(1+\text{exp}\left({\text{x}}_{i}^{T}\hat{\beta }+{\hat{v}}_{i}\right)\right)}^{-1}.$$ (5)

We used an iterative procedure that combines the Penalized Quasi-Likelihood estimation of **β** and **v** = (*v*_1_, …, *v*_*D*_)^*T*^ with REML estimation of $\sigma_{v}^{2}$ to estimate unknown parameters in (4) The MSE estimation of EPP is adopted from Chandra et al. (2011) [44]. Here, the survey weighted probability estimate for a district is modelled as a binomial proportion, with an “effective sample size” that equates the resulting binomial variance to the actual sampling variance of the survey weighted direct estimate for a district [40]. In model (4), the “actual sample size” and “actual sample count” were replaced with the “effective sample size” and “effective sample count” respectively to incorporate the sampling information. For details on model fitting, see S1 File.

### 2.9 Coefficient of variation

The coefficient of variation (CV) of a small area estimate ${\hat{F}}_{\alpha i}$ is computed as $CV\left({\hat{F}}_{\alpha i}\right)=\left(\hat{SE}\left({\hat{F}}_{\alpha i}\right)/{\hat{F}}_{\alpha i}\right)\times 100$, where $\hat{SE}\left({\hat{F}}_{\alpha i}\right)=\sqrt{mse\left({\hat{F}}_{\alpha i}\right)}$ is the estimate of standard error of ${\hat{F}}_{\alpha i}$ and $mse\left({\hat{F}}_{\alpha i}\right)$ is the estimate of $MSE\left({\hat{F}}_{\alpha i}\right)$. It provides a measure of relative error and gives an indication of the precision of the model-based estimates when contrasted with the direct survey estimates. Therefore, estimates are more precise if they have smaller CVs. Conversely, the estimates with larger CVs are considered unreliable and unstable.

### 2.10 SAE validation and benchmarking

In SAE literature, two types of diagnostics are used: the model diagnostics, and the diagnostics for the small area estimates [45]. The former are used to verify the assumptions of the underlying model, i.e., how well the working model performed when it is fitted to data. The latter diagnostics are used to indicate validity and reliability of the small area estimates. We applied 3 diagnostics to our model: (a) the bias diagnostic, (b) the percent coefficient of variation (CV) diagnostic, and (c) the 95% confidence interval diagnostic. Moreover, we benchmarked our small area estimates against the direct estimates at a higher (i.e., state) level. See S1 File for details on diagnostics and benchmarks.

## 3. Results

A generalized linear model for FIP, and linear models for FIG and FIS, were initially fit with 5 auxiliary variables (see Methods). Akaike Information Criterion (AIC) was used for optimal model selection. We identified 2 of these, the proportion of Literacy rate (LR) and the proportion of working population (WP), which contribute significantly to district-specific FI in EIGP. The selected optimal model for FIP as outcome had significant (p<0.01) negative influences from LR and WP with regression coefficients -0.6800 and -2.9125 respectively. The selected optimal models for FIG and FIS as outcomes also had significant (p<0.05) influences from exactly the same 2 auxiliary variables. LR and WP had their respective regression coefficients as -0.0640 and -0.2706 for FIG; and -0.0195 and -0.0803 for FIS. Thus, LR and WP were used as covariates in our model-based SAE analysis. As noted in the Methods, we used empirical predictor of proportion (EPP) for SAE of FIP, and empirical best linear unbiased predictor (EBLUP) for SAE of FIG and FIS. Using a Wald statistic (*W*), we tested the model’s Goodness of Fit [45]. The small values of *W* = 5.68, 15.42, and 25.83 for FIP, FIG, and FIS respectively are significant at 5% level which indicates the consistency between the direct and the model-based SAE results. See S1 File for further details.

As is common for SAE studies, we conducted two types of diagnostics: (a) the model diagnostics, and (b) the diagnostics for the small area estimates [45]. The model diagnostics in S1 Fig show that the normality assumptions were satisfied. The bias diagnostics in S2 Fig indicate that the model-based estimates of FIP, FIG and FIS are less extreme when compared to the corresponding direct estimates, thus demonstrating that the typical SAE outcome shrinks the extreme values towards the average (see S1 File).

The district-specific estimates of the FIP, FIG and FIS along with their CVs and 95% CIs were computed by the direct and SAE methods (see Table 1). The S1 Table lists FIP, FIG and FIS estimates for all 127 rural districts of EIGP. We note in Table 1 that FIP of Bihar (0.62) and UP (0.62) are slightly lower than WB (0.70). Further, the value of FIS (0.03) is the same for each state whereas in WB, FIG (0.13) is marginally higher than FIG (0.11) in both UP and Bihar. The state level estimates shown in Table 1 are reasonably accurate in terms of the CVs. The S2 Table and S3 Fig summarize the district level distribution of sample size (i.e., number of households in sample), the number of food insecure households in sample (sample count) and the sampling fraction in 2011–2012 HCES.

**Table 1:** The estimate and percent coefficient of variation (CV) of FIP, FIG and FIS in EIGP region and the 3 states.

States	Indicators	Estimate	CV
UP	FIP	0.62	12.99
	FIG	0.11	17.39
	FIS	0.03	23.77
Bihar	FIP	0.62	12.60
	FIG	0.11	18.58
	FIS	0.03	24.66
WB	FIP	0.70	10.47
	FIG	0.13	11.46
	FIS	0.03	16.43
EIGP region	FIP	0.64	12.23
	FIG	0.11	16.18
	FIS	0.03	22.11

**Fig 1:**
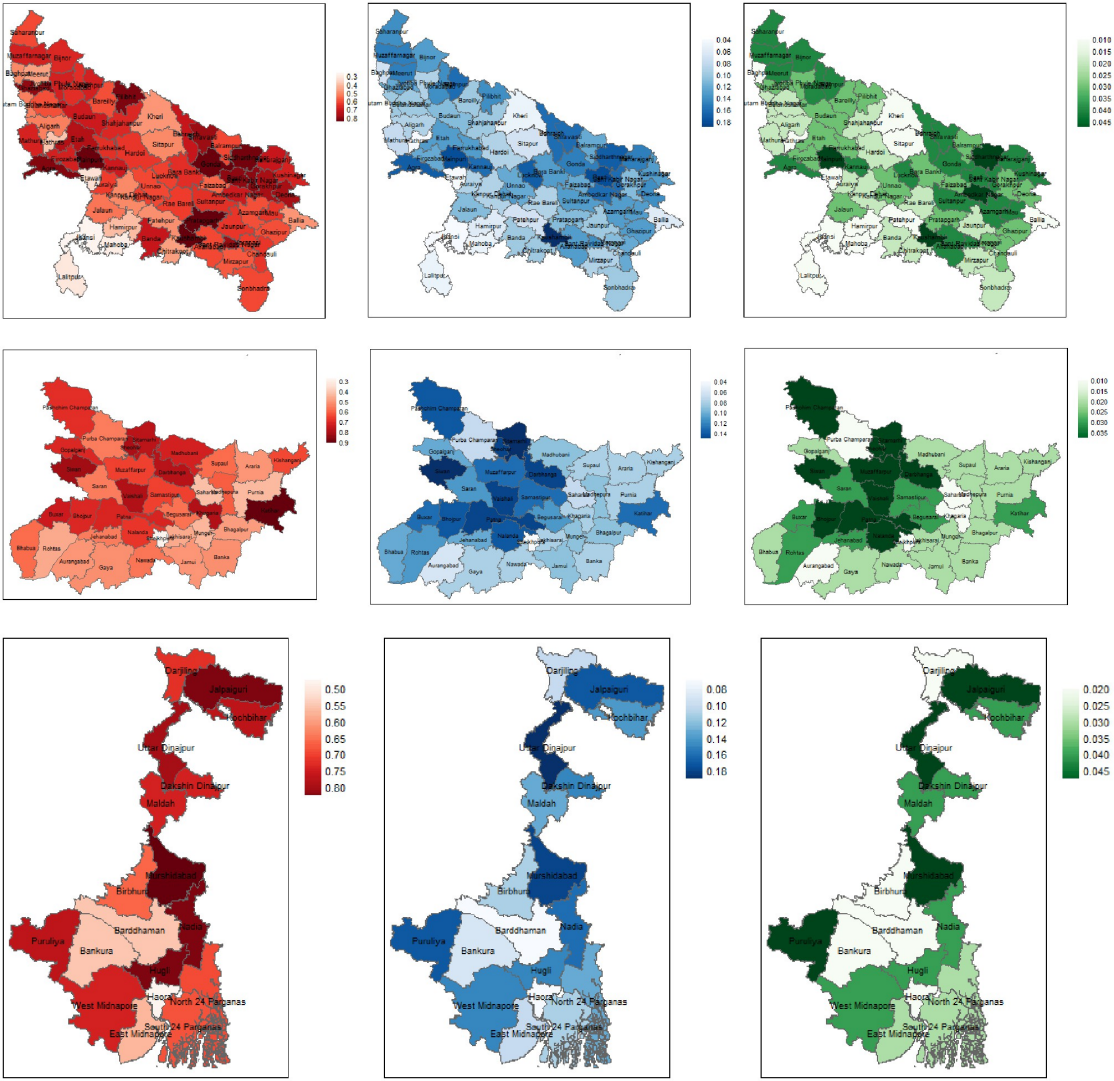
District-wise maps showing the spatial distribution of food insecurity prevalence (left panel), gap (center) and severity (right) generated by SAE method for the states Uttar Pradesh (top), Bihar (middle), and West Bengal (bottom) in the EIGP region. Higher values of food insecurity indicators are shown in darker colors. (Base map source: https://gadm.org/download_country.html).

**Fig 2:**
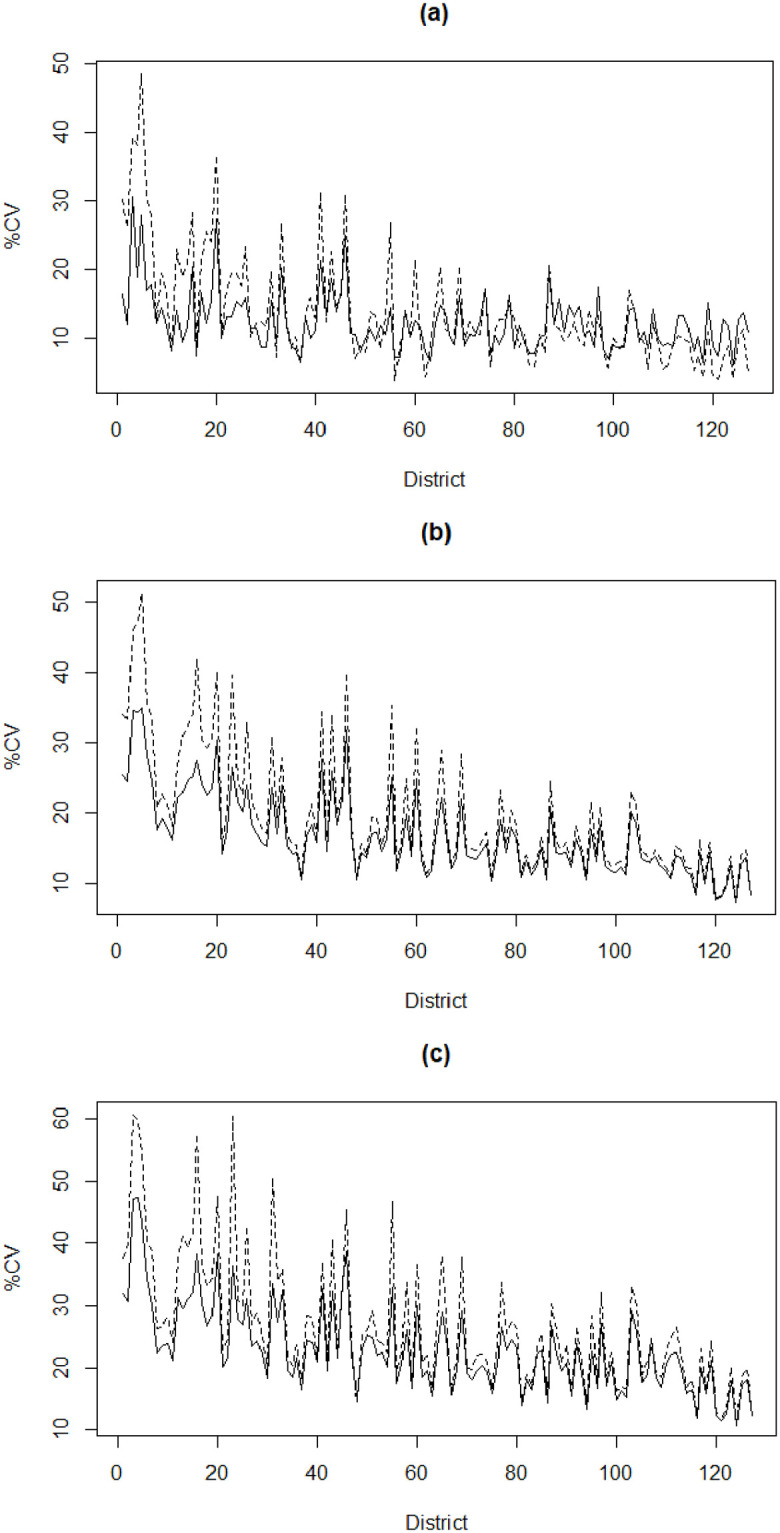
District-wise coefficient of variation (%CV) for the model-based small area estimates (solid line) and the direct estimates (dashed line) of (a) FIP, (b) FIG and (c) FIS. Districts are arranged from left to right in increasing order of sample size.

In Fig 1, we mapped the spatial distributions of FIP, FIG and FIS in UP, Bihar and WB based on our model-based small area estimates. The darker areas on the maps show districts with higher FI. Fig 2 presents the district-wise values of percentage CV for model-based estimates and direct estimates of (a) FIP, (b) FIG and (c) FIS in increasing order of sample sizes. In most districts, the CVs of the model-based estimates are significantly smaller than those of direct survey estimates, thus demonstrating higher precision of the former. Further, such improvement CV is higher for districts with smaller sample sizes as compared to larger sample sizes.

Moreover, a series of diagnostic measures clearly demonstrate that the model-based estimates of FI indicators are more precise than the direct estimates (see S1 File). The district-wise maps of the 95% confidence intervals (CIs) for FIP, FIG and FIS produced by the direct and SAE methods are displayed in S3 Fig. Clearly, the 95% CIs for the direct estimates are wider than the 95% CIs for the model-based estimates. We note that the 95% CI for direct estimates are invalid (for example, values greater than 1.0 for FIP and negative for FIS, etc.) in many districts due to large standard errors.

A set of summary statistics for the direct and model-based estimates along with associated standard errors and CV of the FI indicators (FIP, FIG and FIS) for 127 districts are reported in Table 2. As expected, the averages of the model-based estimates of FI indicators are almost identical to those of the direct estimates but with lower variation. For example, the maximum standard error of FIP estimates generated by the direct and the SAE methods are 0.152 and 0.105 respectively. Moreover, we calculated the ratio of the CVs of the direct and the corresponding model-based estimates for each of FIP, FIG and FIS. The boxplots of these ratios in S4 Fig show that the model-based estimates have CVs that are smaller than those of the direct estimates, and hence are relatively more precise.

**Table 2:** Summary statistics of direct and model-based estimates along with their standard error (SE) and percent coefficient of variation (CV) of FIP, FIG and FIS.

Indicator	Statistics	Direct estimate			Model-based estimate		
		Estimate	SE	CV	Estimate	SE	CV
FIP	Minimum	0.217	0.036	3.97	0.264	0.052	6.23
	1st Quartile	0.532	0.063	8.92	0.548	0.065	9.11
	Median	0.650	0.074	11.40	0.649	0.072	11.45
	Mean	0.635	0.078	13.96	0.635	0.073	12.34
	3rd Quartile	0.743	0.090	17.13	0.733	0.081	14.31
	Maximum	0.912	0.152	48.47	0.903	0.105	30.57
FIG	Minimum	0.032	0.009	7.40	0.036	0.009	7.22
	1st Quartile	0.083	0.016	13.70	0.089	0.015	12.87
	Median	0.119	0.020	16.14	0.117	0.018	14.57
	Mean	0.117	0.020	19.80	0.115	0.018	16.83
	3rd Quartile	0.148	0.024	22.99	0.143	0.021	19.98
	Maximum	0.244	0.038	51.39	0.202	0.027	35.03
FIS	Minimum	0.005	0.002	11.11	0.005	0.002	10.77
	1st Quartile	0.019	0.005	19.78	0.020	0.005	18.17
	Median	0.031	0.007	23.99	0.030	0.006	21.92
	Mean	0.031	0.007	26.79	0.029	0.006	23.02
	3rd Quartile	0.040	0.009	32.59	0.037	0.007	26.86
	Maximum	0.080	0.020	60.70	0.055	0.011	47.30

For benchmarking the reliability of the small area estimates, we aggregated and compared them with estimates at higher spatial levels. Table 3 shows that aggregation of the district-level estimates closely matches the EIGP region and state level estimates of the same FI indicators. The internal benchmarking performance indicated that the model-based estimates do not need to be validated further. An overall geographical distribution of prevalence of FI in the EIGP region, including data from the neighboring country of Bangladesh [26], is interpolated using the estimates and represented as a contour map in S6 Fig.

**Table 3:** Aggregated level estimates of FIP generated by direct and model-based SAE methods in different states and EIGP region.

Indicator	Estimator	EIGP	Uttar Pradesh	Bihar	West Bengal
FIP	Direct	0.653	0.643	0.625	0.702
	Model-based	0.640	0.618	0.621	0.699
FIG	Direct	0.121	0.122	0.108	0.132
	Model-based	0.115	0.113	0.106	0.127
FIS	Direct	0.032	0.033	0.027	0.036
	Model-based	0.028	0.028	0.025	0.033

The SAE results clearly show unequal distribution of FI across the EIGP region (Fig 1). In particular, FIP, FIG and FIS values range from 26.4–90.3% (average 63.5%), 3.6–20.2% (average 11.5%) and 0.5–5.5% (average 2.9%) respectively across the districts of the region. In fact, the intra-state disparities across districts are also noteworthy. FIP across districts in UP, Bihar and WB vary from 47.6–85.7% (62.8%), 27–90.3% (61.2%) and 46.7–84.6% (71%) respectively, where the average is shown in parentheses. The corresponding ranges and averages for FIG are 6.2–20.2% (11.6%), 4.5–16% (10.6%) and 6.8–19.2% (13.2%); and for FIS 1.1–5.5% (2.9%), 0.9–4.3% (2.5%) and 1.6–5.1% (3.4%) in UP, Bihar and WB respectively. We note that the range of disparities in FIP is the highest in Bihar while those for FIG and FIS are the highest in UP.

Interestingly, we observed certain spatial patterns when we focused on the map of each state. In UP, the results indicate an east-west divide in the distribution of FIP, FIG and FIS. For example, in the western part of UP there are many districts with low levels of FIP, FIG and FIS. In contrast, the eastern part of UP has several districts with high rate of FIP, FIG and FIS. The districts with higher percentage of FIP (> 75%) are Mainpuri, Basti, Pratapgarh, Gonda, Kaushambi, Siddharth Nagar, Agra, Sant Ravidas Nagar (currently called Bhadohi), Deoria, Pilibhit, Sant Kabir Nagar, Kannauj, Ghaziabad, and Gorakhpur. In general, FIG and FIS status of these districts are also high except in few cases. For example, although Pilibhit, Ghaziabad and Deoria have high FIP but values of FIG and FIS index of these districts are nominal between 0.14–0.15 and 0.031–0.035 respectively. On the other hand, the UP districts with lower percentage of FIP (< 40%) are Jhansi, Lalitpur, Etawah, Mahoba and Meerut.

In Bihar, except for Sheikhpura (27.5%), FIP exceeds 40% in all other districts. However, only 3 districts—Siwan, Katihar, and Sheohar—have FIP > 80%. Further, high level of FIP exists in many districts from its northern part bordering with Nepal and its western part sharing border with eastern UP, e.g., West Champaran, Sitamarhi and Madhubani in northern Bihar; and Siwan, Buxar and Gopalganj in western Bihar. In WB, as in Bihar, FIP is greater than 50% in all districts except for Howrah (46.7%), which is across the river Ganga from the capital city of Kolkata. Districts with the highest FIP (>80%) are Jalpaiguri, Murshidabad, Nadia, and Hooghly. Notably, Jalpaiguri, in northern WB, which borders Bhutan and Bangladesh in the north and south respectively, and Murshidabad and Nadia in eastern WB, which border Bangladesh, have the highest values of FIP, FIG and FIS in that state. The least vulnerable districts are Howrah, Bankura and Bardhaman (currently split into Paschim Bardhaman and Purba Bardhaman districts). In WB, FI is generally concentrated in the northern and the eastern parts of the state.

**Fig 3:**
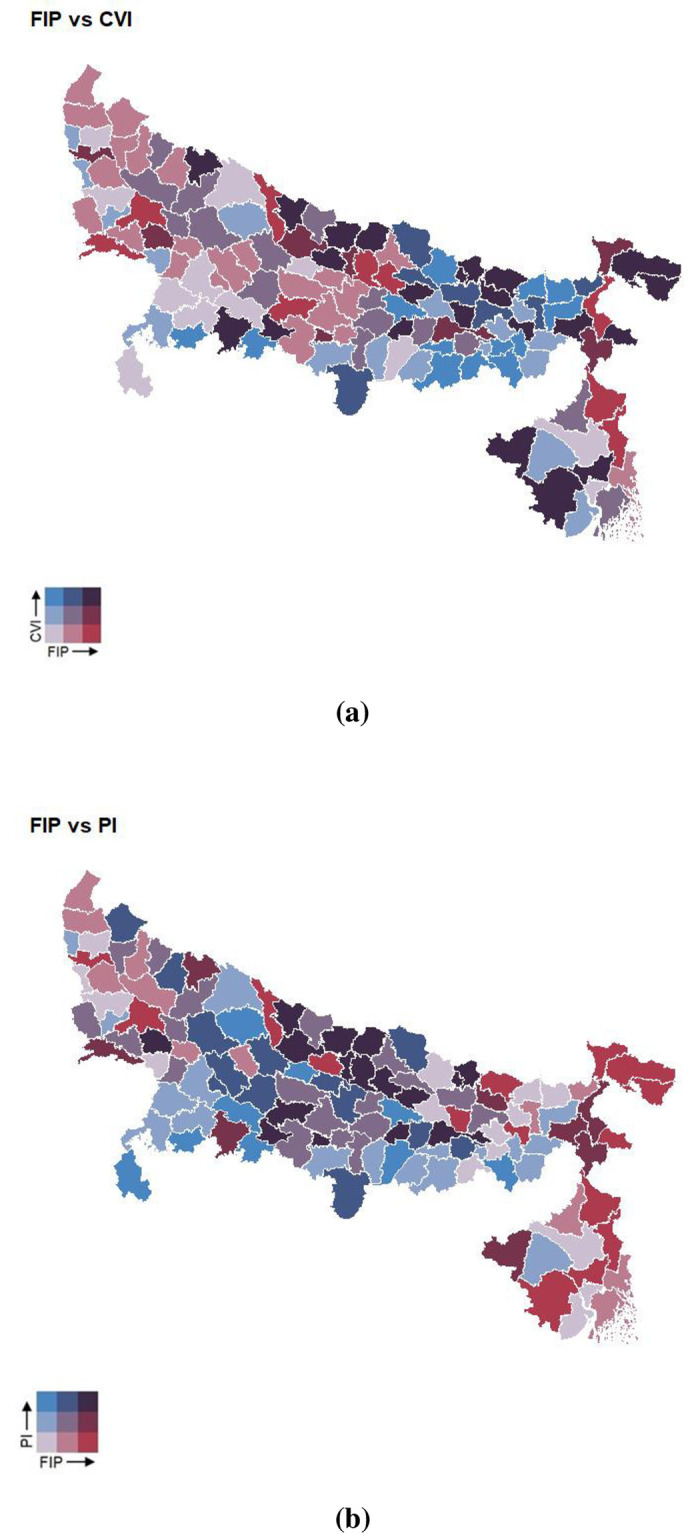
Maps of EIGP showing each district’s FIP against (a) CVI and (b) PI. A bivariate color key is used to depict the range of values for each pair of indices. (Base map source: https://gadm.org/download_country.html).

**Fig 4:**
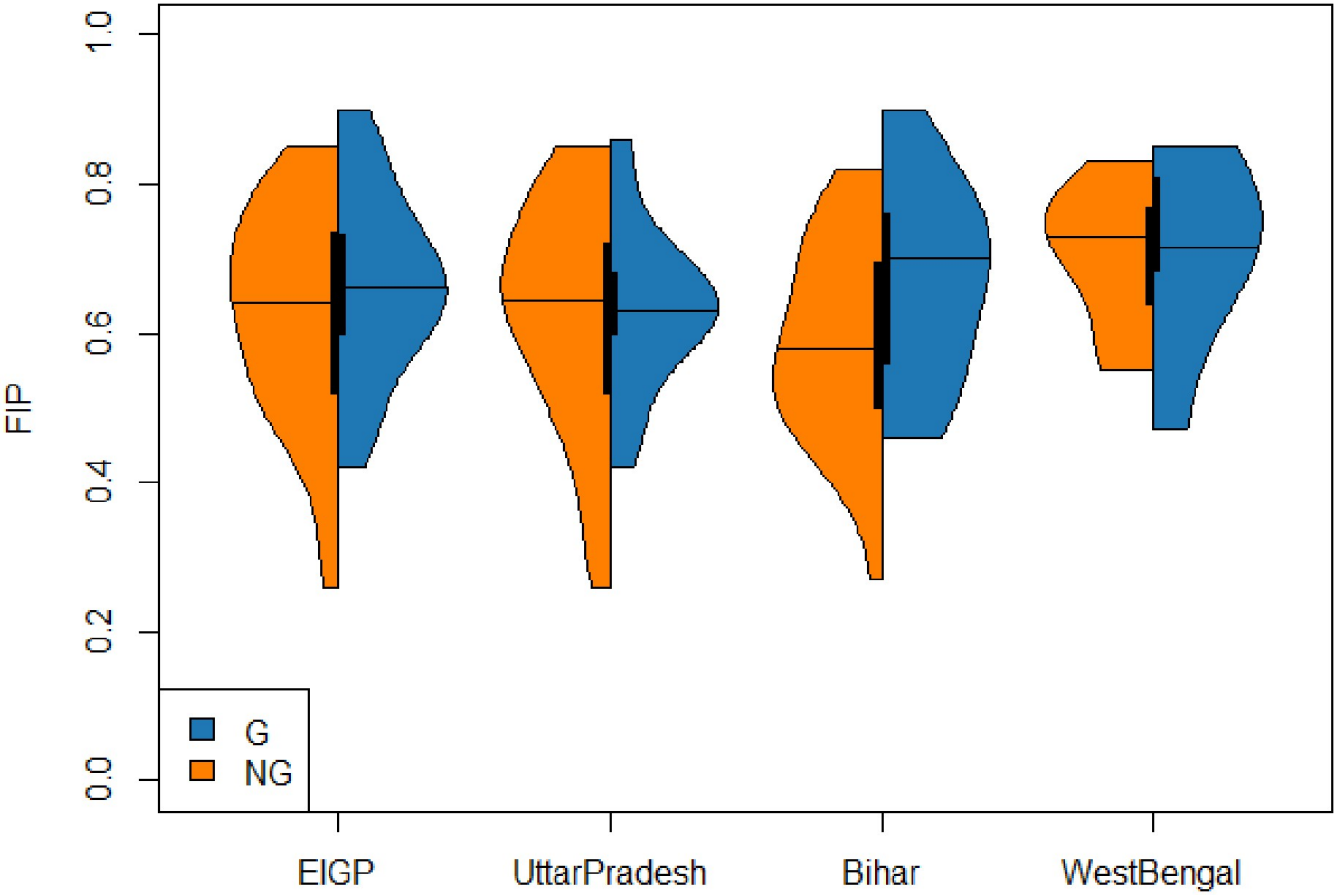
Violinplot for comparing the FIP distributions of the Gangetic (G) and the non-Gangetic (NG) districts of EIGP region and the 3 states. The former (G) are shown in blue and the latter (NG) in orange.

Upon estimation of the district-specific FI indicators, we compared them with the CVI and PI of the same districts. As FIP is relatively independent of CVI and PI (Spearman’s ρ = 0.1 and -0.16 respectively), we depicted FIP using district-wise “bivariate” maps (Fig 3) for comparison with CVI (Fig 3a) and PI (Fig 3b). The red (high FIP) and blue (high CVI) districts exhibited spatial autocorrelation in Fig 3a. Further, we distinguished the districts into Gangetic (i.e., those located on the banks of the river Ganga as well as its major distributaries, Bhagirathi and Hooghly, in WB) and non-Gangetic groups, and compared the distributions of FIP across these 2 groups (Fig 4). While the distributions are similar in the overall region, UP, and WB, interestingly, they vary in the state of Bihar where the median FIP is higher for the Gangetic districts than the non-Gangetic ones.

Notably, we identified several districts that have relatively high values of *both* food insecurity as well as CVI (shaded black in Fig 3a). In fact, some of these also have high PI or low CDI. The resulting shortlist of 22 districts, as shown in Table 4, could be used for further investigation as well as prioritizing the need to address any systemic challenges in such areas via targeted policies that are suited to those conditions (e.g., cropped area, poverty, Gangetic location or otherwise) and requirements of concerned populations.

**Table 4:** A list of 22 districts in EIGP and the state, sample size (Sample), Food Insecurity Prevalence (FIP), Climate Vulnerability Index (CVI), Poverty Index (PI), Cropped Area (in hectares), and Crop Diversity Index (CDI). Group denotes whether a district is Gangetic (G) or non-Gangetic (NG). Other abbreviations: Uttar Pradesh (UP), WB (West Bengal).

State	District	Sample	FIP	CVI	PI	Cropped Area	CDI	Group
Bihar	Katihar	96	0.90	0.725	0.19	253327	4.17	G
Bihar	Sheohar	64	0.80	0.694	0.28	44631	3.35	NG
Bihar	Siwan	88	0.82	0.669	0.28	224847	3.43	NG
Bihar	Sitamarhi	64	0.77	0.668	0.36	233135	2.86	NG
Bihar	Khagaria	64	0.79	0.66	0.12	126037	3.99	G
Bihar	Madhubani	127	0.72	0.659	0.12	NA	NA	NG
Bihar	Buxar	96	0.73	0.656	0.31	201276	3.25	NG
Bihar	Vaishali	96	0.79	0.655	0.12	178780	4.60	G
Bihar	Darbhanga	64	0.78	0.632	0.23	171339	3.50	NG
WB	Cooch Behar	64	0.77	0.681	0.14	257000	3.53	NG
WB	Jalpaiguri	192	0.83	0.679	0.13	203800	4.14	NG
WB	Purulia	128	0.77	0.676	0.26	314507	2.19	NG
WB	Paschim Medinipur	128	0.74	0.653	0.07	284810	0.74	NG
WB	Dakshin Dinajpur	96	0.74	0.649	0.11	186900	4.68	NG
WB	Hooghly	184	0.83	0.627	0.13	210895	2.24	G
UP	Banda	128	0.71	0.659	0.26	474755	4.54	NG
UP	Shravasti	96	0.79	0.653	0.36	191286	2.69	NG
UP	Basti	64	0.81	0.639	0.1	339321	3.41	NG
UP	Pilibhit	64	0.83	0.637	0.19	402836	3.38	NG
UP	Siddharthnagar	32	0.84	0.631	0.3	382895	1.90	NG
UP	Maharajganj	96	0.71	0.636	0.3	369061	2.93	NG
UP	Kaushambi	63	0.86	0.627	0.29	202262	4.83	G

## 4. Discussion

The U.S. Department of Agriculture describes FI as a situation of “limited or uncertain availability of nutritionally adequate and safe foods or limited or uncertain ability to acquire acceptable foods in socially acceptable ways” [46]. This definition draws our attention to the fact that FI is more than just hunger, as is recognized by the 2030 sustainable development goals (SDGs) of the United Nations, and, in particular, SDG2, which pertains to ending hunger, improving food security and nutrition, and promoting sustainable agriculture [47]. Globally, almost 2 billion people face some form of FI. As per the World Food Programme, 135 million people suffer from acute hunger, and the COVID-19 pandemic is expected to double that number [48].

India has the natural resource capacity to achieve nutritional security, reduce health risks and improve environmental sustainability [49]. While the Green Revolution has substantially increased India’s food production over the past half century, its other more mixed outcomes include homogenization of cereal production, unsustainable resource use especially where agroecological conditions are not well-suited, and rising vulnerability to climate variations [20]. The 2022 GHI report specifically notes that “India shows the importance of considering the subnational context when designing programs and policies to target child stunting.” [6]. Yet, in the presence of its inter-regional disparities, India currently lacks the needed disaggregate level data on food security. Towards this, our study used a model-based SAE approach to generate reliable and representative direct and model-based estimates. The resulting maps illustrate unequal spatial distributions of FI prevalence, gap and severity among the populations in different districts of UP, Bihar and WB in EIGP region.

Our models were informed by various district-specific covariates, among which the literacy rate (LR) and the proportion of working population (WP) had significant negative influences respectively on a district’s food insecurity. Interestingly, while the share of LR in the EIGP is lower than the national average, other recent, independent studies on malnutrition in India also provide evidence in the same direction [50]. In small area literature [23], two standard types of validation are conducted: internal and external. We have executed both types of procedures thoroughly, and validated the model outputs using several diagnostic measures. They revealed significant gains in precision by our small area approach in obtaining the district-level estimates of FI. Since the area level model is developed at the district level by incorporating the household level variation as the sampling error variance, the model may not capture unknown variability due to any mid-level hierarchical components (such as a village or a sub-district area) in between a household and a district. Such misspecification is an important issue for understated standard errors of model-based estimator in the literature of unit level SAE method for poverty estimation [41, 42].

EIGP has long been studied for its relatively low productivity, poor infrastructure, limited capacity for private investment, and climate sensitivity [51]. Multiple effects of climate change, including recurrent droughts, floods, and cyclones, are causes of serious concern in EIGP [52]. Notably, UP, Bihar and WB appeared among the 10 most climatically vulnerable states in a recently published report [14]. Additionally, UP, Bihar and WB also rank (5, 9, and 14 respectively) among the poorest states in India [13]. Thus, both climate vulnerability and poverty might act as stressors that can exacerbate the prevailing FI, and disparities therein, among different populations in these states. On the other hand, crop diversity, especially of an agricultural district, might confer it with local resiliency and adaptability against both climate change as well as FI. Hence, we also computed crop diversity and poverty indices for each district in EIGP. Taken together with our estimates of FI measures, they provide a nuanced understanding of the overall conditions in the region.

The COVID-19 pandemic has impacted food security and livelihoods in many parts of the world [53, 54]. This is especially true for India which reported the second highest number of COVID-19 cases of any country in the world (>32 million) by mid-2021, with UP, Bihar and WB together contributing to almost 4 million cases [55]. Recommendations to address the ensuing FI range from expansion of governmental support for existing welfare schemes to investment in targeted social protection strategies and safety nets [56, 57]. Our SAE can provide disaggregated data for identifying the districts in EIGP with quantifiable impacts on their food security, as the diagnostic measures clearly demonstrate that our model-based estimates of FI indicators are more precise than direct estimates. For instance, relatively higher FIP in the Gangetic districts, as we observed in Bihar and WB, may benefit from systems that are resilient to FI while being adaptive to the stresses of climate change. In fact, while the number of Gangetic districts may be less than non-Gangetic ones, the policies to mitigate risks in the two groups should preferably be distinctive—with due consideration to their respective extreme event scenarios, and focused on adaptation strategies that are suited to each group and sustained implementation [19].

The Gangetic basin is considered to be under extreme pressure and one of the most vulnerable regions in terms of climate change [52]. Recurrent floods and droughts, extreme heat and seismic events, river bank erosion and sediment dynamics, urbanization and pollution, livelihoods and internal migration, land use and crop management, etc.–a variety of determinants would need to be considered for any integrated data-driven policy framework to ensure food security in EIGP. Towards this, data such as from the present study could provide spatially detailed insights to existing mechanisms, e.g., the Member of Parliament Local Area Development Scheme in India. For instance, post-pandemic assessment of the impact on population-specific food insecurity may lead to creation of durable community assets as well as support for micro, small and medium enterprises (or MSMEs [58]) in the vulnerable districts to jointly address local unemployment, poverty and food insecurity. The SAE approach allows us to take a closer look in that strategic direction as we plan to extend it to studies of the other states and regions in our future work. The inclusion of data for both urban and rural districts collected from multiple rounds of survey can lead to the development of a powerful and robust spatio-temporal area level model and, thus, better understanding of this complex phenomenon.

## 5. Conclusion

We estimated the district-specific food insecurity indicators, and mapped their local disparities over the EIGP region. By comparing food insecurity with indicators of climate vulnerability, poverty and crop diversity, we shortlisted the vulnerable districts in EIGP. These estimates and maps can be effective for informed policy-making to build local resiliency and address systemic vulnerabilities where they matter most in the post-pandemic era.

## Supporting information

S1 Table District wise estimates of FIP, FIG, FIS, and other variables. (DOCX)

S2 Table District level household summary in 2011–2012 HCES data. (DOCX)

S1 Fig District level residuals, histograms and q-q plots. (DOCX)

S2 Fig Bias diagnostic plot of small area versus direct estimates. (DOCX)

S3 Fig District-wise sampling fractions for the 3 EIGP states. (DOCX)

S4 Fig District-wise confidence interval plots. (DOCX)

S5 Fig Coefficient of variation boxplot of small area versus direct estimates. (DOCX)

S6 Fig Geographical distribution of FIP in EIGP region. (DOCX)

S1 File (DOCX)

## Abbreviations

AIC: Akaike Information Criterion
CDI: Crop Diversity Index
CI: Confidence Interval
CV: Coefficient of Variation
CVI: Climate Vulnerability Index
DST: Department of Science & Technology
EBLUP: Empirical Best Linear Unbiased Predictor
EIGP: Eastern Indo-Gangetic Plains
EPP: Empirical Predictor of Proportion
FAO: Food and Agriculture Organization
FI: Food Insecurity
FIG: Food Insecurity Gap
FIP: Food Insecurity Prevalence
FIS: Food Insecurity Severity
HCES: Household Consumption Expenditure Survey
LLMM: Logistic Linear Mixed Model
MSME: Micro, Small and Medium enterprise
NSSO: National Sample Survey Office
SAE: Small Area Estimation
SDC: Swiss Agency for Development & Cooperation
SDGs: Sustainable Development Goals
ST: Scheduled Tribe
UP: Uttar Pradesh
WB: West Bengal
WP: Working Population
