# Supplementary material for: Food insecurity in the Eastern Indo-Gangetic plain: Taking a closer look
Source: PLoS One. 2023 Jan 5;18(1):e0279414. doi: 10.1371/journal.pone.0279414 (PMC9815573; doi:10.1371/journal.pone.0279414)
Supplement: S1 Table — (DOCX) [file pone.0279414.s001.docx]

**S1 Table. District wise estimates of FIP, FIG, FIS, and other variables.**

List of 127 rural districts in EIGP, state, latitude, longitude, Food Insecurity Prevalence (FIP), Food Insecurity Gap (FIG), Food Insecurity Severity (FIS), Climate Vulnerability Index (CVI), Poverty Index (PI). Abbreviations: Uttar Pradesh (UP), WB (West Bengal).

| **No.** | **State** | **District** | **Latitude** | **Longitude** | **FIP** | **FIG** | **FIS** | **CVI** | **PI** |
| --- | --- | --- | --- | --- | --- | --- | --- | --- | --- |
| 1 | Bihar | Katihar | 25.88 | 87.97 | 0.90 | 0.13 | 0.03 | 0.725 | 0.19 |
| 2 | Bihar | Araria | 26.22 | 87.78 | 0.50 | 0.08 | 0.02 | 0.707 | 0.1 |
| 3 | Bihar | Kishanganj | 27.32 | 88.55 | 0.64 | 0.08 | 0.02 | 0.707 | 0.12 |
| 4 | Bihar | Purnia | 26.3 | 87.78 | 0.43 | 0.08 | 0.02 | 0.701 | 0.26 |
| 5 | Bihar | Jamui | 25.53 | 86.37 | 0.50 | 0.09 | 0.02 | 0.7 | 0.34 |
| 6 | Bihar | Sheohar | 26.85 | 85.5 | 0.80 | 0.15 | 0.04 | 0.694 | 0.28 |
| 7 | Bihar | Madhepura | 26.52 | 87.3 | 0.60 | 0.09 | 0.02 | 0.68 | 0.06 |
| 8 | Bihar | East Champaran | 27.08 | 85.52 | 0.55 | 0.07 | 0.01 | 0.673 | 0.14 |
| 9 | Bihar | Lakhisarai | 25.28 | 87.5 | 0.46 | 0.08 | 0.02 | 0.672 | 0.16 |
| 10 | Bihar | Siwan | 26.37 | 84.6 | 0.82 | 0.16 | 0.04 | 0.669 | 0.28 |
| 11 | Bihar | Sitamarhi | 27 | 85.8 | 0.77 | 0.16 | 0.04 | 0.668 | 0.36 |
| 12 | Bihar | Khagaria | 25.83 | 86.78 | 0.79 | 0.08 | 0.01 | 0.66 | 0.12 |
| 13 | Bihar | Gopalganj | 26.47 | 84.43 | 0.70 | 0.10 | 0.02 | 0.659 | 0.2 |
| 14 | Bihar | Madhubani | 26.58 | 86.13 | 0.72 | 0.09 | 0.02 | 0.659 | 0.12 |
| 15 | Bihar | Buxar | 25.93 | 84.63 | 0.73 | 0.13 | 0.03 | 0.656 | 0.31 |
| 16 | Bihar | Vaishali | 26.42 | 86.3 | 0.79 | 0.15 | 0.04 | 0.655 | 0.12 |
| 17 | Bihar | Supaul | 26.55 | 86.42 | 0.58 | 0.08 | 0.02 | 0.655 | 0.09 |
| 18 | Bihar | Arwal | 25.4 | 85.12 | 0.73 | 0.12 | 0.03 | 0.648 | 0.19 |
| 19 | Bihar | Gaya | 25.25 | 85 | 0.51 | 0.08 | 0.02 | 0.647 | 0.19 |
| 20 | Bihar | Muzaffarpur | 26.12 | 85.45 | 0.65 | 0.13 | 0.04 | 0.643 | 0.23 |
| 21 | Bihar | Nawada | 24.88 | 85.58 | 0.56 | 0.08 | 0.02 | 0.639 | 0.16 |
| 22 | Bihar | West Champaran | 27.33 | 84.83 | 0.69 | 0.14 | 0.04 | 0.633 | 0.33 |
| 23 | Bihar | Darbhanga | 26.28 | 86.5 | 0.78 | 0.15 | 0.04 | 0.632 | 0.23 |
| 24 | Bihar | Munger | 25.38 | 86.5 | 0.44 | 0.09 | 0.02 | 0.627 | 0.25 |
| 25 | Bihar | Jehanabad | 25.25 | 85 | 0.55 | 0.10 | 0.03 | 0.626 | 0.26 |
| 26 | Bihar | Samastipur | 25.92 | 85.08 | 0.65 | 0.13 | 0.03 | 0.623 | 0.18 |
| 27 | Bihar | Aurangabad | 24.75 | 84.42 | 0.50 | 0.06 | 0.01 | 0.622 | 0.19 |
| 28 | Bihar | Nalanda | 25.33 | 85.87 | 0.67 | 0.14 | 0.04 | 0.621 | 0.28 |
| 29 | Bihar | Banka | 25.47 | 87.52 | 0.51 | 0.08 | 0.02 | 0.619 | 0.22 |
| 30 | Bihar | Bhojpur | 25.92 | 85.1 | 0.70 | 0.14 | 0.04 | 0.618 | 0.35 |
| 31 | Bihar | Saharsa | 26.47 | 87 | 0.42 | 0.07 | 0.01 | 0.616 | 0.11 |
| 32 | Bihar | Sheikhpura | 25.22 | 86.42 | 0.27 | 0.04 | 0.01 | 0.612 | 0.17 |
| 33 | Bihar | Begusarai | 25.7 | 86.22 | 0.57 | 0.11 | 0.03 | 0.611 | 0.09 |
| 34 | Bihar | Patna | 25.62 | 85.22 | 0.71 | 0.15 | 0.04 | 0.609 | 0.29 |
| 35 | Bihar | Saran | 26.52 | 85.25 | 0.55 | 0.11 | 0.03 | 0.607 | 0.16 |
| 36 | Bihar | Bhagalpur | 25.25 | 87.02 | 0.49 | 0.09 | 0.02 | 0.605 | 0.18 |
| 37 | Bihar | Kaimur | 25.67 | 84.02 | 0.58 | 0.10 | 0.02 | 0.601 | 0.22 |
| 38 | Bihar | Rohtas | 25.58 | 84.17 | 0.46 | 0.11 | 0.03 | 0.585 | 0.31 |
| 39 | WB | Cooch Behar | 26 | 89.5 | 0.77 | 0.14 | 0.04 | 0.681 | 0.14 |
| 40 | WB | Jalpaiguri | 26.5 | 88.7 | 0.83 | 0.17 | 0.05 | 0.679 | 0.13 |
| 41 | WB | Purulia | 23 | 86.4 | 0.77 | 0.17 | 0.05 | 0.676 | 0.26 |
| 42 | WB | Paschim Medinipur | 22.4 | 87.38 | 0.74 | 0.15 | 0.04 | 0.653 | 0.07 |
| 43 | WB | Dakshin Dinajpur | 25.37 | 88.55 | 0.74 | 0.15 | 0.04 | 0.649 | 0.11 |
| 44 | WB | Hooghly | 22.9 | 88.3 | 0.83 | 0.15 | 0.04 | 0.627 | 0.13 |
| 45 | WB | Malda | 25 | 88.1 | 0.74 | 0.13 | 0.04 | 0.622 | 0.17 |
| 46 | WB | Purba Medinipur | 21.94 | 87.77 | 0.57 | 0.10 | 0.03 | 0.621 | 0.16 |
| 47 | WB | Bankura | 24.4 | 87.2 | 0.55 | 0.09 | 0.02 | 0.615 | 0.19 |
| 48 | WB | South 24 Parganas | 22.13 | 88.4 | 0.68 | 0.11 | 0.03 | 0.614 | 0.11 |
| 49 | WB | Birbhum | 23.7 | 87.7 | 0.66 | 0.11 | 0.02 | 0.609 | 0.1 |
| 50 | WB | Darjeeling | 27.1 | 88.3 | 0.72 | 0.10 | 0.02 | 0.607 | 0.16 |
| 51 | WB | Uttar Dinajpur | 25.98 | 88.05 | 0.80 | 0.19 | 0.05 | 0.583 | 0.24 |
| 52 | WB | Nadia | 23.4 | 88.5 | 0.83 | 0.16 | 0.04 | 0.573 | 0.16 |
| 53 | WB | Bardhaman | 23.2 | 87.9 | 0.55 | 0.07 | 0.02 | 0.553 | 0.1 |
| 54 | WB | Howrah | 22.6 | 88 | 0.47 | 0.07 | 0.02 | 0.547 | 0.16 |
| 55 | WB | North 24 Parganas | 22.61 | 88.4 | 0.69 | 0.13 | 0.03 | 0.532 | 0.09 |
| 56 | WB | Murshidabad | 24.1 | 88.3 | 0.85 | 0.18 | 0.05 | 0.531 | 0.16 |
| 57 | UP | Ballia | 25.73 | 84.18 | 0.48 | 0.06 | 0.01 | 0.676 | 0.44 |
| 58 | UP | Banda | 25.33 | 80.37 | 0.71 | 0.10 | 0.02 | 0.659 | 0.26 |
| 59 | UP | Shravasti | 27.85 | 82.15 | 0.79 | 0.16 | 0.04 | 0.653 | 0.36 |
| 60 | UP | Sonbhadra | 24.68 | 83.92 | 0.61 | 0.10 | 0.02 | 0.64 | 0.38 |
| 61 | UP | Basti | 26.8 | 82.77 | 0.81 | 0.18 | 0.04 | 0.639 | 0.1 |
| 62 | UP | Pilibhit | 28.63 | 79.85 | 0.83 | 0.14 | 0.03 | 0.637 | 0.19 |
| 63 | UP | Mahoba | 27.15 | 83.57 | 0.29 | 0.05 | 0.01 | 0.626 | 0.35 |
| 64 | UP | Siddharth Nagar | 27.5 | 83.15 | 0.84 | 0.17 | 0.05 | 0.631 | 0.3 |
| 65 | UP | Chitrakoot | 25.22 | 80.8 | 0.46 | 0.10 | 0.02 | 0.63 | 0.29 |
| 66 | UP | Kheri | 27.9 | 80.8 | 0.48 | 0.05 | 0.01 | 0.571 | 0.24 |
| 67 | UP | Maharajganj | 25.3 | 79.92 | 0.71 | 0.15 | 0.04 | 0.636 | 0.3 |
| 68 | UP | Shahjahanpur | 27.9 | 79.95 | 0.67 | 0.10 | 0.02 | 0.622 | 0.23 |
| 69 | UP | Gonda | 27.47 | 82.02 | 0.85 | 0.15 | 0.04 | 0.621 | 0.28 |
| 70 | UP | Sant Ravidas Nagar | 25.33 | 83.47 | 0.79 | 0.14 | 0.04 | 0.619 | 0.38 |
| 71 | UP | Sant Kabir Nagar | 26.72 | 83.07 | 0.81 | 0.16 | 0.04 | 0.619 | 0.32 |
| 72 | UP | Mirzapur | 25.17 | 82.62 | 0.58 | 0.09 | 0.02 | 0.615 | 0.25 |
| 73 | UP | Ghaziabad | 28.67 | 77.47 | 0.80 | 0.12 | 0.03 | 0.614 | 0.06 |
| 74 | UP | Bara Banki | 26.93 | 81.22 | 0.66 | 0.13 | 0.03 | 0.61 | 0.43 |
| 75 | UP | Hardoi | 27.38 | 80.17 | 0.64 | 0.09 | 0.02 | 0.608 | 0.29 |
| 76 | UP | Balrampur | 27.42 | 82.17 | 0.61 | 0.12 | 0.03 | 0.606 | 0.23 |
| 77 | UP | Kannauj | 27.05 | 79.97 | 0.70 | 0.16 | 0.04 | 0.565 | 0.16 |
| 78 | UP | Baghpat | 28.93 | 77.23 | 0.50 | 0.07 | 0.01 | 0.603 | 0.25 |
| 79 | UP | Sitapur | 25.18 | 80.87 | 0.50 | 0.07 | 0.01 | 0.6 | 0.32 |
| 80 | UP | Kanpur Dehat | 26.47 | 80.4 | 0.50 | 0.09 | 0.02 | 0.517 | 0.26 |
| 81 | UP | Mainpuri | 27.23 | 79.05 | 0.85 | 0.18 | 0.05 | 0.596 | 0.34 |
| 82 | UP | Gautam Buddha Nagar | 28.95 | 77.53 | 0.52 | 0.10 | 0.02 | 0.596 | 0.07 |
| 83 | UP | Chandauli | 25.45 | 83.45 | 0.64 | 0.12 | 0.03 | 0.595 | 0.21 |
| 84 | UP | Etawah | 26.78 | 79.03 | 0.33 | 0.04 | 0.01 | 0.595 | 0.16 |
| 85 | UP | Budaun | 28.03 | 79.17 | 0.70 | 0.11 | 0.03 | 0.593 | 0.16 |
| 86 | UP | Farrukhabad | 27.4 | 79.62 | 0.65 | 0.13 | 0.03 | 0.593 | 0.19 |
| 87 | UP | Raebareli | 26.23 | 81.27 | 0.63 | 0.09 | 0.02 | 0.592 | 0.36 |
| 88 | UP | Mau | 25.95 | 83.6 | 0.64 | 0.13 | 0.04 | 0.591 | 0.2 |
| 89 | UP | Jalaun | 26.13 | 79.38 | 0.54 | 0.11 | 0.03 | 0.581 | 0.22 |
| 90 | UP | Rampur | 28.8 | 79.08 | 0.69 | 0.16 | 0.04 | 0.589 | 0.23 |
| 91 | UP | Ghazipur | 25.57 | 83.58 | 0.60 | 0.13 | 0.03 | 0.586 | 0.25 |
| 92 | UP | Sultanpur | 26.25 | 82.07 | 0.65 | 0.13 | 0.04 | 0.585 | 0.22 |
| 93 | UP | Bareilly | 28.37 | 79.45 | 0.60 | 0.10 | 0.03 | 0.585 | 0.44 |
| 94 | UP | Hamirpur | 25.97 | 80.2 | 0.41 | 0.06 | 0.01 | 0.584 | 0.2 |
| 95 | UP | Bijnor | 29.38 | 79.18 | 0.67 | 0.13 | 0.04 | 0.584 | 0.51 |
| 96 | UP | Azamgarh | 26.05 | 83.22 | 0.60 | 0.13 | 0.04 | 0.581 | 0.32 |
| 97 | UP | Firozabad | 27.15 | 78.4 | 0.69 | 0.12 | 0.03 | 0.581 | 0.19 |
| 98 | UP | Jaunpur | 25.77 | 82.73 | 0.64 | 0.10 | 0.02 | 0.531 | 0.19 |
| 99 | UP | Deoria | 26.38 | 83.7 | 0.77 | 0.14 | 0.03 | 0.578 | 0.32 |
| 100 | UP | Agra | 27.17 | 78.08 | 0.82 | 0.17 | 0.04 | 0.572 | 0.18 |
| 101 | UP | Kushinagar | 27.23 | 84.47 | 0.68 | 0.13 | 0.04 | 0.525 | 0.26 |
| 102 | UP | Etah | 27.58 | 78.67 | 0.75 | 0.13 | 0.03 | 0.568 | 0.14 |
| 103 | UP | Hathras | 27.95 | 78.03 | 0.39 | 0.04 | 0.01 | 0.591 | 0.24 |
| 104 | UP | Kanpur Nagar | 27.8 | 78.63 | 0.62 | 0.13 | 0.03 | 0.548 | 0.43 |
| 105 | UP | Varanasi | 25.33 | 83 | 0.63 | 0.11 | 0.03 | 0.563 | 0.17 |
| 106 | UP | Fatehpur | 25.92 | 80.87 | 0.49 | 0.07 | 0.01 | 0.557 | 0.45 |
| 107 | UP | Auraiya | 24.45 | 79.22 | 0.63 | 0.09 | 0.02 | 0.549 | 0.21 |
| 108 | UP | Kaushambi | 25.53 | 81.37 | 0.86 | 0.20 | 0.05 | 0.627 | 0.29 |
| 109 | UP | Saharanpur | 29.97 | 77.38 | 0.61 | 0.14 | 0.04 | 0.547 | 0.09 |
| 110 | UP | Ambedkar Nagar | 26.43 | 52.55 | 0.70 | 0.16 | 0.05 | 0.546 | 0.3 |
| 111 | UP | Allahabad | 25.47 | 81.9 | 0.61 | 0.13 | 0.04 | 0.539 | 0.24 |
| 112 | UP | Faizabad | 26.78 | 82.2 | 0.56 | 0.12 | 0.03 | 0.536 | 0.3 |
| 113 | UP | Unnao | 26.8 | 80.72 | 0.61 | 0.12 | 0.03 | 0.532 | 0.5 |
| 114 | UP | Jhansi | 25.45 | 78.62 | 0.26 | 0.04 | 0.01 | 0.605 | 0.22 |
| 115 | UP | Muzaffarnagar | 29.47 | 77.73 | 0.69 | 0.15 | 0.04 | 0.531 | 0.08 |
| 116 | UP | Bahraich | 27.57 | 81.63 | 0.71 | 0.16 | 0.04 | 0.527 | 0.09 |
| 117 | UP | Aligarh | 27.5 | 79.67 | 0.52 | 0.09 | 0.02 | 0.526 | 0.16 |
| 118 | UP | Lalitpur | 24.37 | 78.47 | 0.31 | 0.05 | 0.01 | 0.494 | 0.33 |
| 119 | UP | Moradabad | 28.85 | 78.82 | 0.66 | 0.14 | 0.04 | 0.521 | 0.15 |
| 120 | UP | Kanshiram Nagar | 27.8 | 78.64 | 0.42 | 0.08 | 0.02 | 0.517 | 0.16 |
| 121 | UP | Mathura | 27.47 | 77.68 | 0.68 | 0.10 | 0.02 | 0.513 | 0.19 |
| 122 | UP | Bulandshahar | 28.4 | 77.9 | 0.60 | 0.11 | 0.03 | 0.512 | 0.11 |
| 123 | UP | Pratapgarh | 25.57 | 81.98 | 0.86 | 0.17 | 0.04 | 0.509 | 0.4 |
| 124 | UP | Lucknow | 26.92 | 80.98 | 0.62 | 0.11 | 0.03 | 0.568 | 0.08 |
| 125 | UP | Gorakhpur | 26.75 | 83.4 | 0.77 | 0.15 | 0.04 | 0.487 | 0.28 |
| 126 | UP | Meerut | 29.02 | 77.75 | 0.46 | 0.09 | 0.03 | 0.48 | 0.06 |
| 127 | UP | Jyotiba Phule Nagar | 28.9 | 78.52 | 0.69 | 0.12 | 0.03 | NA | 0.18 |
