# Supplementary material for: Food insecurity in the Eastern Indo-Gangetic plain: Taking a closer look
Source: PLoS One. 2023 Jan 5;18(1):e0279414. doi: 10.1371/journal.pone.0279414 (PMC9815573; doi:10.1371/journal.pone.0279414)
Supplement: S2 Table — (DOCX) [file pone.0279414.s002.docx]

**S2 Table. District level household summary in 2011-2012 HCES data.**

| States | Summary | Sample size | Sample count | Sampling fraction |
| --- | --- | --- | --- | --- |
| UP | Minimum | 32 | 10 | 0.00015 |
|  | Average | 83 | 53 | 0.00023 |
|  | Maximum | 128 | 111 | 0.00032 |
|  |  |  |  |  |
| Bihar | Minimum | 64 | 15 | 0.00013 |
|  | Average | 87 | 49 | 0.00024 |
|  | Maximum | 128 | 98 | 0.00074 |
|  |  |  |  |  |
| WB | Minimum | 64 | 45 | 0.00020 |
|  | Average | 128 | 128 | 0.00024 |
|  | Maximum | 236 | 236 | 0.00033 |
|  |  |  |  |  |

District level summary of distribution of number of households in sample (sample size), number of food insecure households in sample (sample count) and the sampling fraction in 2011-2012 HCES data.
