# Supplementary material for: Food insecurity in the Eastern Indo-Gangetic plain: Taking a closer look
Source: PLoS One. 2023 Jan 5;18(1):e0279414. doi: 10.1371/journal.pone.0279414 (PMC9815573; doi:10.1371/journal.pone.0279414)
Supplement: S1 Fig — (DOCX) [file pone.0279414.s003.docx]

**S1 Figure. District level residuals, histograms and q-q plots.**


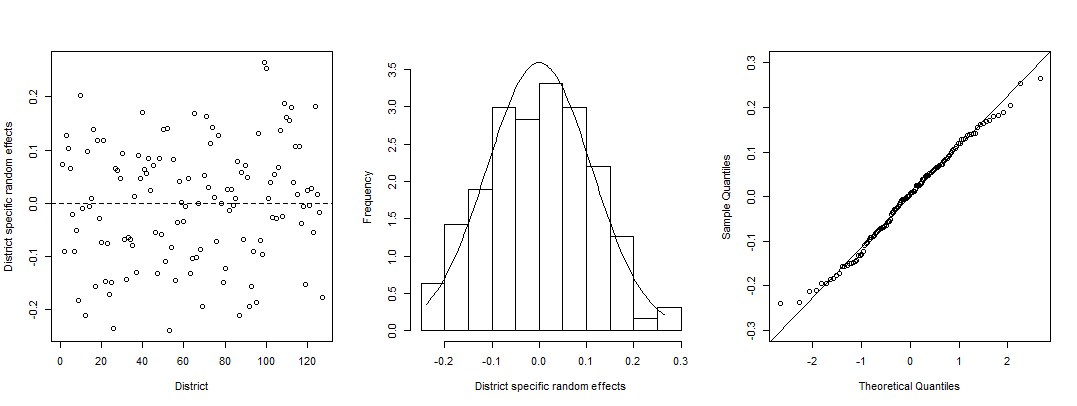

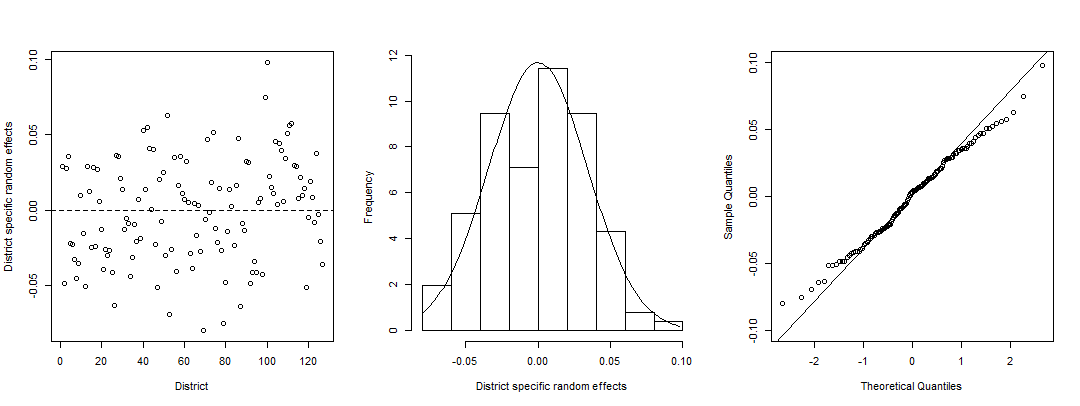


**
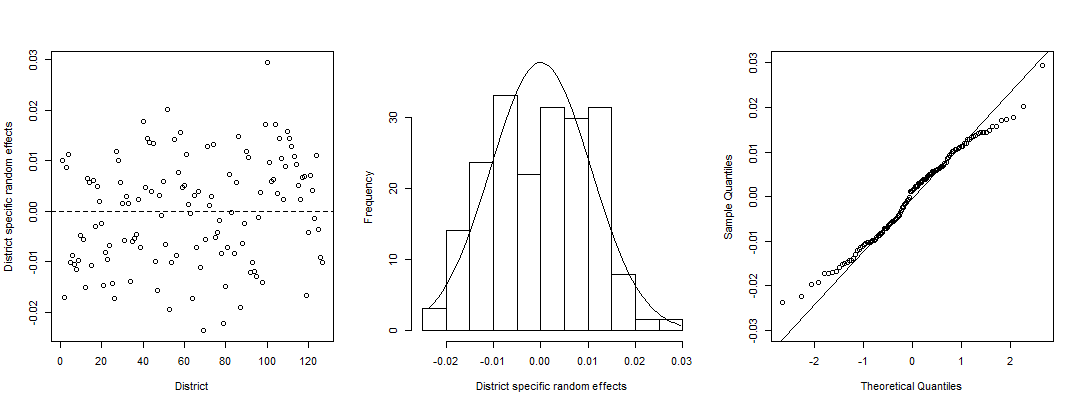
**

Distributions of the district level residuals (left plots), histograms of the district level residuals (center plots) and normal q-q plots of the district level residuals (right plots) for FIP, FIG and FIS (top, middle, and bottom panels respectively).
