# Supplementary material for: Food insecurity in the Eastern Indo-Gangetic plain: Taking a closer look
Source: PLoS One. 2023 Jan 5;18(1):e0279414. doi: 10.1371/journal.pone.0279414 (PMC9815573; doi:10.1371/journal.pone.0279414)
Supplement: S2 Fig — (DOCX) [file pone.0279414.s004.docx]

**S2 Figure. Bias diagnostic plot of small area versus direct estimates.**


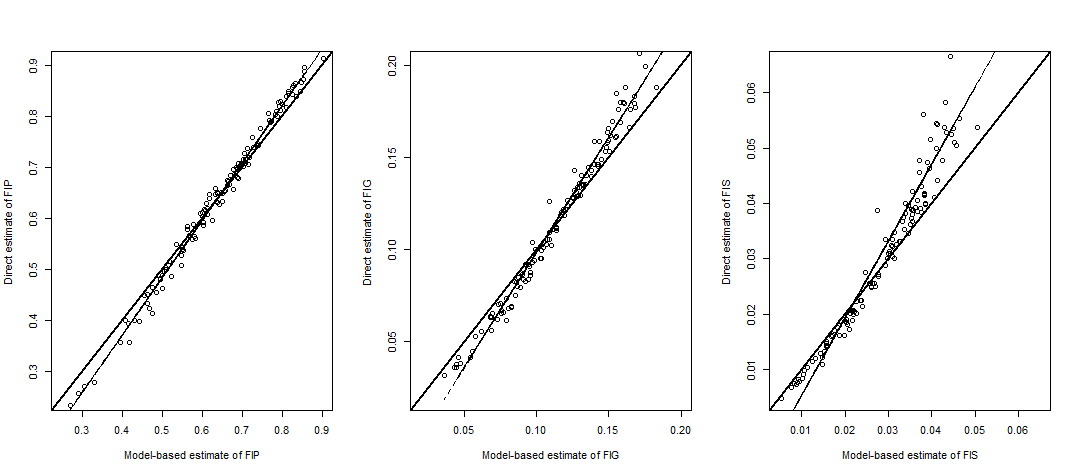


Bias diagnostic plot with *y* = *x* line (solid) and regression line (dotted) for FIP, FIG and FIS estimates: model-based small area versus direct estimates.
