# Supplementary material for: Food insecurity in the Eastern Indo-Gangetic plain: Taking a closer look
Source: PLoS One. 2023 Jan 5;18(1):e0279414. doi: 10.1371/journal.pone.0279414 (PMC9815573; doi:10.1371/journal.pone.0279414)
Supplement: S3 Fig — (DOCX) [file pone.0279414.s005.docx]

**S3 Figure. District-wise sampling fractions for the 3 EIGP states.**

**
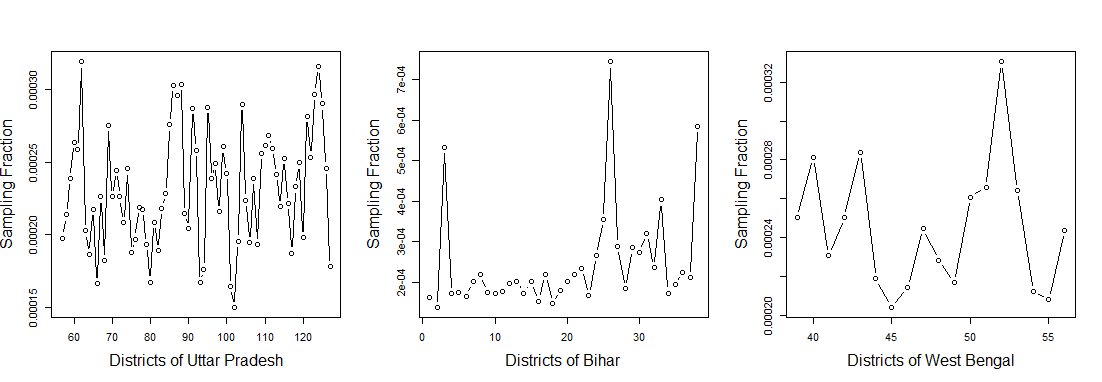
**

District-wise distributions of the sampling fractions for UP (left), Bihar (center), and WB (right).
