# Supplementary material for: Food insecurity in the Eastern Indo-Gangetic plain: Taking a closer look
Source: PLoS One. 2023 Jan 5;18(1):e0279414. doi: 10.1371/journal.pone.0279414 (PMC9815573; doi:10.1371/journal.pone.0279414)
Supplement: S4 Fig — (DOCX) [file pone.0279414.s006.docx]

**S4 Figure**. **District-wise confidence interval plots.**


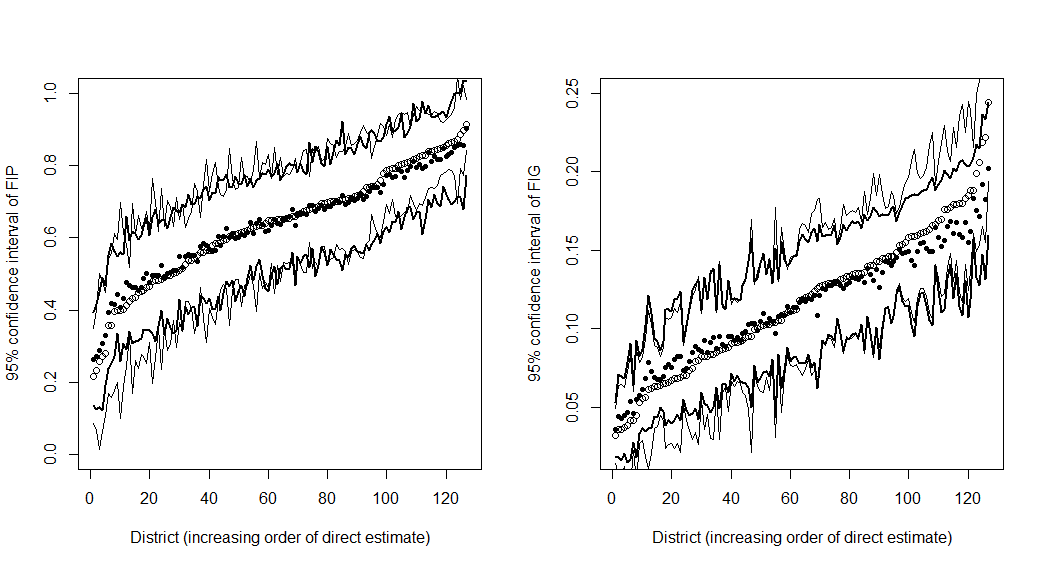
**
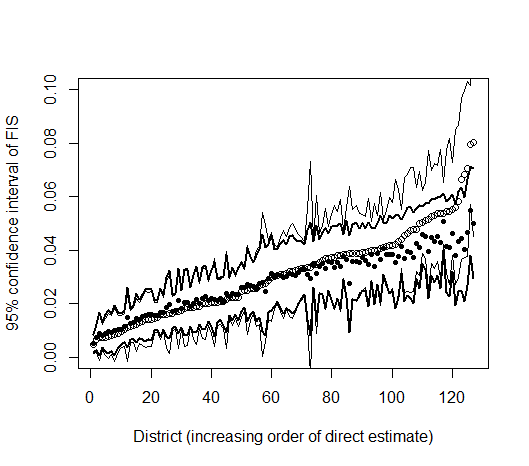
**

District-wise 95 percent nominal confidence interval (95% CI) for the direct (thin line) and SAE (solid line) methods. Direct (dotted points) and model-based estimates (solid points) for the food insecurity indicators (FIP – above left plot; FIG – above right; FIS – bottom) are shown with their 95% CI.
