# Supplementary material for: Food insecurity in the Eastern Indo-Gangetic plain: Taking a closer look
Source: PLoS One. 2023 Jan 5;18(1):e0279414. doi: 10.1371/journal.pone.0279414 (PMC9815573; doi:10.1371/journal.pone.0279414)
Supplement: S5 Fig — (DOCX) [file pone.0279414.s007.docx]

**S5 Figure. Coefficient of variation boxplot of small area versus direct estimates.**


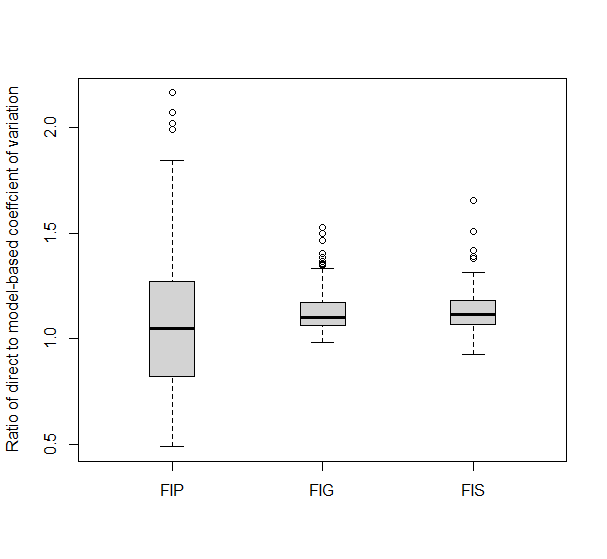


Boxplot of the distribution of the coefficient of variation (CV) of the model-based estimates compared with direct estimates of FIP, FIG and FIS.
