# Supplementary material for: Food insecurity in the Eastern Indo-Gangetic plain: Taking a closer look
Source: PLoS One. 2023 Jan 5;18(1):e0279414. doi: 10.1371/journal.pone.0279414 (PMC9815573; doi:10.1371/journal.pone.0279414)
Supplement: S6 Fig — (DOCX) [file pone.0279414.s008.docx]

**S6 Figure.** **Geographical distribution of FIP in EIGP region.**


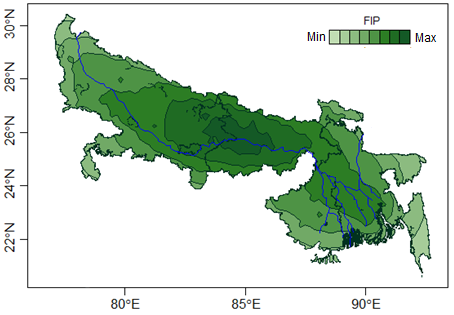


Geographical distribution of FIP in EIGP region including Bangladesh. The Indian states of UP, Bihar, and WB along with the neighboring country of Bangladesh (using SAE data from Hossain et al., 2020) are depicted. In addition, the two major river systems in EIGP, the Ganga and the Brahmaputra, are shown. (Base map source: <https://gadm.org/download_country.html>)
