# Supplementary material for: Food insecurity in the Eastern Indo-Gangetic plain: Taking a closer look
Source: PLoS One. 2023 Jan 5;18(1):e0279414. doi: 10.1371/journal.pone.0279414 (PMC9815573; doi:10.1371/journal.pone.0279414)
Supplement: S1 File — (DOCX) [file pone.0279414.s009.docx]

**Supplementary Materials (SM)**

**Food Insecurity in the Eastern Indo-Gangetic Plain: Taking a Closer Look**

Saumyadipta Pyne*, Saurav Guha, Sumonkanti Das, Meghana Ray and Hukum Chandra.

*Corresponding author: Saumyadipta Pyne (spyne@ucsb.edu)

**Fitting of SAE Models:** We fit a generalized linear model between district-specific direct estimates of Food Insecurity Prevalence (FIP) and a set of 4 auxiliary district-specific variables: Proportion of scheduled caste population, Proportion of scheduled tribe population (ST), Literacy rate(LR), and Proportion of working population (WP). The auxiliary data for these 71 (UP) + 38 (Bihar) + 18 (WB) = 127 districts were obtained from the 2011 Population Census of India. We used the glm() function in R, with the family parameter as “binomial” and the district specific sample sizes as weight. The final model for FIP include two auxiliary variables, LRand WP, based on the optimal model selection criterion, Akaike Information Criterion (AIC), which attained a value of 1796. For this model, null deviance is 1246.8 on 126 degrees of freedom and including the two auxiliary variables (LR and WP) has decreased the deviance to 1183.5 on 122 degrees of freedom, a significant reduction in deviance. The residual deviance has reduced by 63.3 with a loss of four degrees of freedom. We also used Hosmer-Lemeshow goodness of fit test to examine the fitted model (i.e., model fits depends on the difference between the model and the observed data) using the hoslem.test() function in R. The p-value of Hosmer Lemeshow goodness of fit test was 0.9979, which indicated that the model fit well with no significant difference between the model and the observed data (i.e., the p-value > 0.05).

We fit linear models between district-specific direct estimates of Food Insecurity Gap (FIG) and Food Insecurity Severity (FIS) and the set of 4 auxiliary variables as mentioned above using the lm() function in R and specifying the district specific sample sizes as the weight. Again, LR and WP are identified as two auxiliary variables that are significant (p <0.05) in the final selected models fitted for both FIG as well as FIS. The regression coefficients of LR and WP are respectively -0.06404and -0.27064for FIG, and -0.019585 and -0.080354for FIS. Thus, we used LR and WP as the district-specific covariates for our small area estimation of FIP, FIG and FIS. The EPP (equation 5 in the Methods section of the paper) was used for computing the district-wise small area estimates of FIP, and the EBLUP (equation 3 in the Methods section of the paper) for the same for FIG and FIS.

**Goodness-of-Fit diagnostic:** We used this to check whether the differences $D_{i}=\hat{F}_{\alpha i}^{Direct}-\hat{F}_{\alpha i}$ between direct and model-based estimates over all districts $i$ are statistically different. The null hypothesis is that the direct and model-based estimates are statistically equivalent. The test statistic, equivalent to a Wald test, is computed as $W=\sum_{i} \left\{D_{i}^{2}\left[var\text{(}\hat{F}_{\alpha i}^{Direct}\text{)}+mse\text{(}\hat{F}_{\alpha i}\text{)}\right]^{-1}\right\}$. Under the assumption that $\hat{F}_{\alpha i}^{Direct}$ and $\hat{F}_{\alpha i}$ are independently distributed, which is not unreasonable for large sample sizes, the value of $W$ can be compared with an appropriate critical value from a chi square distribution with degrees of freedom *D* equal to the number of districts, which is 127. Note, for Goodness-of-Fit (GoF) analysis, *D* = 127 gives a critical value of 154.302 at 5% level of significance. A small value (<154.302 here) of $W$ indicates no statistically significant difference between the model-based and the direct estimates. The values of $W$ for the model-based estimates of FIP, FIG and FIS are 5.68, 15.42 and 25.83 respectively. Each of these values are smaller than the 154.302, which indicates that our model-based small area estimates are consistent with the direct estimates.

**SAE Diagnostics:** In small area applications, two types of diagnostics are used: the model diagnostics, and the diagnostics for the small area estimates. The former diagnostics are used to verify the assumptions of the underlying model, while the latter diagnostics are used to provide an indication of validity and reliability of the small area estimates.

In the small area models described by equations (2) and (4) in the Methods section of the paper, the random area (district-specific) effects are assumed to have a normal distribution with mean zero and fixed variance $\sigma_{u}^{2}$. If the model assumptions are satisfied, then the district level residuals are expected to be randomly distributed around zero. We used histograms and q-q plots to inspect the normality assumption. Supplementary Figure S1 displays the distributions of the district level residuals (left plots), histograms (center plots), and normal q-q plots of the district level residuals (right plots) for FIP, FIG and FIS (top to bottom plots). The plots in Supplementary Figure S1 show that the district level residuals are randomly distributed around zero. The histograms and the q-q plots also provide evidence in support of the normality assumption.

We also used the Shapiro-Wilk test (implemented using the shapiro.test function in R) to examine the normality of the district random effects. The Shapiro-Wilk test with p-value lower than 0.05 was used to test if the data deviated from normality. The values of Shapiro-Wilk test statistics for the district level residuals, each with 127 degrees of freedom, are 0.989, 0.992 and 0.984 and p-values 0.437, 0.694 and 0.127 for models fitted with FIP, FIG and FIS respectively. In each case, the Shapiro-Wilk p-value was larger than 0.05, and hence, the district random effects are likely to be normally distributed. The model diagnostics measures clearly show that the normality assumptions were satisfied reasonably well with the data that we have used in our SAE analysis.

We applied three commonly used diagnostics measures for assessing the validity and the reliability of the model-based estimates of the food insecurity indicators (FIP, FIG and FIS): (a) the bias diagnostic, (b) the percent coefficient of variation (CV) diagnostic, and (c) the 95% confidence interval (CI) diagnostic. The first diagnostic evaluates the validity while the last two determine the improved precision of the model-based small area estimates. The CV is described in the main paper. Below we describe the bias diagnostic and the CI.

**Bias diagnostic:** The basic idea underpinning the bias diagnostic is that since direct estimates are unbiased, their regression on the true values should be linear and correspond to the identity ($y=x$) line. If the model-based estimates are “close” to these true values, then the regression of the direct estimates on these model-based estimates should be similar. We therefore plotted the direct estimates (*Y*-axis) against the corresponding model-based estimates (*X*-axis), and looked for divergence of the fitted least squares regression line (shown by dashed line) from the $Y=X$ line (solid line). The bias diagnostic plots in Supplementary Figure S2 clearly indicate that the model-based estimates of FIP, FIG and FIS are less extreme when compared to the corresponding direct estimates, demonstrating that the typical SAE outcome shrinks the extreme values towards the average. The values of adjusted *R*^2^ for the fitted regression line between the direct estimates and the model-based estimates for FIP, FIG, and FIS are 99.2, 97.9 and 91.9 percent respectively. Similarly, high Pearson’s correlation coefficients between the direct and the model-based estimates (0.996 for FIP, 0.989 for FIG and 0.958 for FIS) also support their consistency.

**The 95% confidence interval (CI) diagnostic:** We compared the width of the CIs for the direct estimates compared to the model-based estimates. For the more precise estimates, we expect the width to be narrower. In addition, we consider the *coverage* diagnostic to assess the validity of the CIs generated by the model-based SAE methods. The 95% CIs for the direct estimates should contain the “truth” approximately 95% of the time. This should also hold for the CIs surrounding the model-based estimates. We adjusted both sets of intervals, so that their chance of overlapping is 95% and counted how often they did overlap. Assuming that the estimated coverage of the direct CIs is correct, comparing the counts to the binomial distribution provides a non-parametric significance test of the bias of model estimates relative to their precision. First, we first obtained adjusted 95% CIs for the direct and model-based estimates using the critical values given by $c=1.96\left(\sqrt{var\text{(}\hat{F}_{\alpha i}^{Direct}\text{)}+mse\text{(}\hat{F}_{\alpha i}\text{)}}\right)\left(\sqrt{var\text{(}\hat{F}_{\alpha i}^{Direct}\text{)}}+\sqrt{mse\text{(}\hat{F}_{\alpha i}\text{)}}\right)^{-1}$. Then, we counted the number of times the intervals did not overlap, which should be approximately 5%.(1)

**Benchmarking:** The model-based small area estimates were aggregated to higher levels (EIGP region and state) at which they were then compared with the direct estimates at the same levels. Let $\hat{F}_{\alpha i}$ and $N_{i}$ denote the small area estimate of a food insecurity indicator $F_{\alpha i}$ and the population size for district *i*. Then the region and state-level estimates of the food insecurity indicator $F_{\alpha}$ are calculated as $\hat{F}_{\alpha}=\sum_{i=1}^{D} N_{i}\hat{F}_{\alpha i}/\sum_{i=1}^{D} N_{i}$,$\alpha=0,1\text{ and 2}$.

**Poverty Index (PI) estimation:** Let the population *U* of size *N* be due to *D* districts. The population unit in district *i*, $i=1,...,D$, is represented by $U_{i}$, with population size $N_{i}$, such that $U=\bigcup_{i=1}^{D} U_{i}$ and $N=\sum_{i=1}^{D} N_{i}$. We used the subscript *s* and *r* to denote the units belong to the sampled and non-sampled parts in the population along with the sample size $n_{i}$ for district *i*. For the sampled part, let $s=\bigcup_{i=1}^{D} s_{i}$ and $n=\sum_{i=1}^{D} n_{i}$where denotes the units in the sample in district *i.* We assume that is the value of a binary target variable for unit *j* in district *i* and our aim is to estimate the small area population counts $y_{i}=\sum_{j\in U_{i}} y_{ij}$or the small area proportions $P_{i}=N_{i}^{-1}\left(\sum_{j\in U_{i}} y_{ij}\right)$. We denote the $y_{si}=\sum_{j\in s_{i}} y_{ij}$as the sample count in district *i*. Area-specific *p*-vector auxiliary variables denoted by $x_{i}$ is available from secondary data sources such as the census. A $D$-vector district-specific random effects $\mathbf{u}=\left(u_{1},\ldots,u_{D}\right)^{T}$ where $u_{i}\sim N\left(0, \sigma_{u}^{2}\right)$,$i=1,...,D$. In line with Johnson *et al*. (2010)(2) and Chandra *et al*. (2011)(3), when the aim is to estimate small area proportion, then the $y_{si} \sim\text{Bin}(n_{i},\pi_{i})$ where $\pi_{i}$ is the probability of prevalence in area *i*. the GLMM with logistic model linking $\pi_{i}$ with the covariates $x_{i}$ is then

$logit(\pi_{i})=\ln\left\{\pi_{i}(1-\pi_{i})^{-1}\right\}=\eta_{i}=\mathbf{x}_{i}^{T}\beta+u_{i}$, with $\pi_{i}=\exp(\eta_{i})\left\{1+\exp(\eta_{i})\right\}^{-1}=\mathrm{expit}(\eta_{i})$.

Under this model, the mean of $y_{si}$ given $u_{i}$ is $\mu_{si}=E\left(y_{si} | u_{i}\right) =n_{i} \mathrm{expit}(\mathbf{x}_{i}^{T}\beta+u_{i})$. Without loss of generality and put $\pi=(\pi_{1},...,\pi_{D})^{T}$ and $X=\text{(}x_{1}^{T},....,x_{D}^{T})^{T}$. Aggregating different district level models leads to the population level model as

$$g(\pi)=\eta=X\beta+u$$

where $g(\pi)=(g(\pi_{1}),...,g(\pi_{D}))^{T}$ and $\eta=(\eta_{1},...,\eta_{D})^{T}$. A plug-in empirical predictor (EP) of the population count $y_{i}$ in district *i* is

$\hat{y}_{i}^{EP}=y_{si} + \hat{\mu}_{ri}=y_{si} + \left(N_{i} -n_{i}\right)\hat{\pi}_{i}^{EP}$,

where $\hat{\pi}_{i}^{EP}=\mathrm{expit}(\hat{\eta}_{i})$ for a binary target variable with $\hat{\eta}_{i}=\mathbf{x}_{i}^{T}\hat{\beta}+\hat{u}_{i}$. An estimate of the corresponding proportion or rate in area *i* is obtained as $\hat{P}_{i}^{EP}=N_{i}^{-1}\hat{y}_{i}^{EP}$**.** For areas with zero sample sizes (i.e., non-sampled areas), the conventional approach to estimating area proportions or counts is synthetic estimation, based on a suitable GLMM fitted to the counts from the sampled areas.(4) For a non-sampled district *i* with associated vector of covariates $x_{i,out}$, $\hat{\eta}_{i}=x_{i,out}^{T}\hat{\beta}$ and the synthetic estimator of $y_{i}$ is given by $\hat{y}_{i}^{SYN}=N_{i}\hat{\pi}_{i}^{SYN}$, with $\hat{\pi}_{i}^{SYN}=\mathrm{expit}(\hat{\eta}_{i})$ for a binary target variable. By letting $\hat{y}_{i}$ denote the total number of households below the poverty line in district $i$, we computed the poverty index (PI) as the proportion $\hat{P}_{i}$ of poor households in that district.

**Crop Diversity Index:** Following Jost (2006)(5), we computed the crop diversity index (CDI) of a district as the exponent of the Shannon diversity index (*H*) i.e., $CDI= e^{H}$, where $H= -\sum_{j=1}^{n} p_{j}\ln p_{j}$ and $p_{j}$ is the proportion of the total cropped area under crop species $j$ in that district. In each state, we considered $n=$ 9-10 major crops (in terms of cropped area as per available land use data) for this analysis.

**References**

1. Brown G, Chambers R, Heady P, Heasman D. Evaluation of small area estimation methods - an application to unemployment estimates from the UK LFS. Proc Stat Canada Symp 2001 Achiev Data Qual a Stat Agency A Methodol Perspect. 2001;(January):1–13.

2. Johnson FA, Chandra H, Brown JJ, Padmadas SS. District-level estimates of institutional births in ghana: Application of small area estimation technique using census and DHS data. J Off Stat. 2010;26(2):341–59.

3. Chandra H, Chambers R, Salvati N. Small area estimation of proportions in business surveys. J Stat Comput Simul [Internet]. 2012 Jun 1 [cited 2021 Jun 2];82(6):783–95. Available from: https://ro.uow.edu.au/infopapers/2018

4. Chandra H, Salvati N, Sud UC. Disaggregate-level estimates of indebtedness in the state of Uttar Pradesh in India: an application of small-area estimation technique. http://dx.doi.org/101080/026647632011559202 [Internet]. 2011 Nov [cited 2021 Aug 22];38(11):2413–32. Available from: https://www.tandfonline.com/doi/abs/10.1080/02664763.2011.559202

5. Jost L. Entropy and diversity. Oikos [Internet]. 2006 May 1 [cited 2021 Aug 22];113(2):363–75. Available from: https://onlinelibrary.wiley.com/doi/full/10.1111/j.2006.0030-1299.14714.x
